# *Wdr26* insufficiency causes Skraban-Deardorff syndrome–like neurodevelopmental deficits in mice

**DOI:** 10.1172/JCI195537

**Published:** 2026-05-15

**Authors:** Xingyun Xu, Yaohui Zhou, Shiyao Xu, Hongjie Zhou, Xuexia Lin, Yuhao Luo, Yu Xu, Zhigang Miao, Wei Ge, Hao Yang, Xingshun Xu

**Affiliations:** 1 Institute for Fetology, The First Affiliated Hospital of Soochow University, Suzhou, China.; 2 Institute of Neuroscience, Soochow University, Suzhou, China.; 3 Department of Neurology, The Affiliated Suzhou Hospital of Nanjing Medical University, Suzhou, China.; 4 Department of Neurology, The Affiliated Hospital of Xuzhou Medical University, Xuzhou, China.; 5 Suzhou Key Laboratory of Brain Diseases, Suzhou, China.

**Keywords:** Development, Neuroscience, Neurodevelopment, Neurological disorders, Ubiquitin-proteosome system

## Abstract

Skraban-Deardorff syndrome, a rare neurodevelopmental disorder caused by WD repeat domain 26 (*WDR26*) haploinsufficiency, is characterized by intellectual disability, seizures, autistic-like behaviors, and craniofacial anomalies. Despite its genetic association with variants disrupting the C-terminal to LisH (CTLH) E3 ubiquitin ligase complex, the molecular mechanisms linking *WDR26* dysfunction to neurodevelopmental deficits remain unclear. Here, we demonstrate that *Wdr26* heterozygous-KO mice (*Wdr26^+/–^*) recapitulated core clinical features of the syndrome, including learning and memory impairments, social dysfunction, heightened seizure susceptibility, and motor deficits, alongside rare craniofacial and dental abnormalities. Mechanistically, *Wdr26* haploinsufficiency stabilized RUNX1 translocation partner 1 (RUNX1T1), a transcriptional coactivator critical for neuronal differentiation, by impairing its ubiquitination and proteasomal degradation, consequently disrupting the level of microtubule-associated protein 2 (MAP2), a key regulator of dendritic architecture and synaptic plasticity. Early intervention in neonatal *Wdr26^+/–^* mice (P0.5) using AAV-shRNA–mediated *Runx1t1* knockdown reversed MAP2 overexpression and behavioral deficits. Notably, the antipsychotic risperidone ameliorated cognitive and social impairments in *Wdr26^+/–^* mice by upregulating WDR26 levels, suggesting a potential therapeutic avenue. Our findings not only establish the animal model as a robust preclinical tool but also define the WDR26/RUNX1T1/MAP2 regulatory axis as pivotal to the syndrome’s pathogenesis, while identifying actionable therapeutic targets.

## Abstract 2

Skraban-Deardorff syndrome, caused by WDR26 deficiency, impairs protein breakdown, leading to neurodevelopmental issues; reducing Runx1t1 or risperidone treatment alleviates symptoms in mice, highlighting potential therapies.

## Introduction

Skraban-Deardorff syndrome, a rare autosomal dominant disorder identified in 2017, is primarily characterized by intellectual disability (1). To date, 33 cases have been documented in the literature, of which 3 remain clinically undescribed (2–4), and 30 cases have been described in detail (1, 5–11). The syndrome is associated with a unique set of phenotypic features, including intellectual disability, developmental delay, autistic tendencies, seizures, gait abnormalities, hypotonia, infant feeding difficulties, distinctive facial features, and skeletal abnormalities (1, 5–11). Currently, there is a lack of effective clinical treatments for Skraban-Deardorff syndrome, imposing a substantial economic and psychological burden on both the patients and their families. The disorder is attributed to variants or microdeletions in the WD repeat domain 26 (*WDR26*) gene, located on chromosome 1q42.12, which result in haploinsufficiency. Skraban et al. demonstrated that the frameshift variants of *WDR26* are correlated with decreased mRNA and protein levels (1). Thus, the development of Skraban-Deardorff syndrome is directly linked to the expression of the WDR26. Despite growing awareness of WDR26’s involvement in the syndrome, further research is needed to elucidate the precise molecular mechanisms by which *WDR26* haploinsufficiency leads to the disease phenotypes.

The *WDR26* gene encodes a subunit of the E3 ubiquitin ligase multiprotein complex known as C-terminal to LisH (CTLH), targeting specific proteins for degradation (12–17). WDR26 serves as a structural scaffold for the CTLH E3 complex, facilitating the assembly of large, hollow, supramolecular CTLH E3 assemblies (18–22). Recent research has suggested that these supramolecular CTLH E3 assemblies recognize substrates via WDR26 (19, 23, 24). In addition, it has been documented that the interaction between WDR26 and muscadine, another subunit of the CTLH E3 complex, plays a role in regulating the E3 ligase activity of the CTLH complex (20). Importantly, mutations in *WDR26* have been demonstrated to disrupt the binding of the CTLH E3 complex to its substrates (25). Therefore, the impaired function of the CTLH E3 complex, resulting from variants or microdeletions in the *WDR26* gene, markedly contributes to the pathological mechanism of Skraban-Deardorff syndrome, primarily due to the functional imbalance in the ubiquitin-proteasome system.

In this study, we examined the phenotypes of *Wdr26*-KO mice (*Wdr26^–/–^*) and found that the WDR26 deficiency adversely affected embryonic development, resulting in embryonic lethality in *Wdr26^–/–^* mice. *Wdr26^+/–^* mice exhibited behavioral abnormalities associated with Skraban-Deardorff syndrome, including impairments in learning and memory, diminished social interaction, heightened seizure susceptibility, motor dysfunction, and rare craniofacial and dental developmental abnormalities. Further studies revealed that *Wdr26* haploinsufficiency increased the levels and stability of RUNX1T1 (also known as myeloid translocation genes 8 [MTG8]) proteins through ubiquitination; indeed, reducing RUNX1T1 levels in the cortex ameliorated cognitive and socialization deficits in *Wdr26^+/–^* mice. Notably, we identified that risperidone elevated WDR26 levels, thereby enhancing cognitive performance and socialization in *Wdr26^+/–^* mice. This study provides insights into the underlying disease mechanisms and potential therapeutic strategies.

## Results

### Generation of Wdr26-deficient mice.

**Figure 1:**
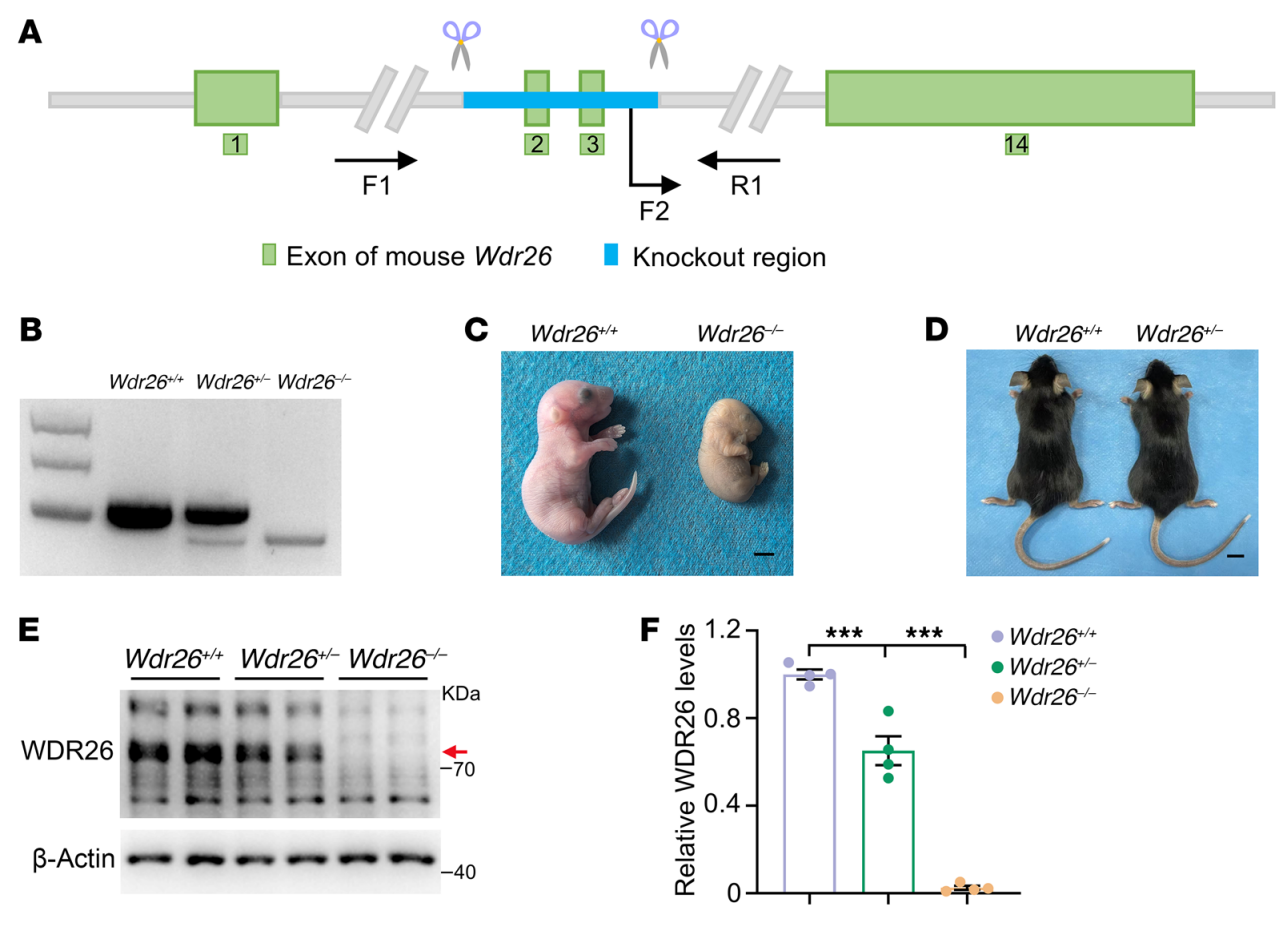
*Wdr26^+/–^* mice are established and verified. (**A**) The illustration showed the KO region of *Wdr26*. (**B**) Genomic DNA from the brains of *Wdr26^+/+^*, *Wdr26^+/–^*, and *Wdr26^–/–^* mouse embryos were examined by PCR. (**C**) *Wdr26^+/+^* and *Wdr26^–/–^* E17.5 embryos. Scale bar: 2 mm. (**D**) Two-month-old *Wdr26^+/+^* and *Wdr26^+/–^* mice. Scale bar: 1 cm. Data in **B**–**D** are representative of 3 independent experiments. (**E**) WDR26 levels were tested by Western blotting in the brains of *Wdr26^+/+^*, *Wdr26^+/–^*, and *Wdr26^–/–^* mouse embryos at E12.5. The red arrow indicates the position of the WDR26 band. (**F**) Quantitative analysis of WDR26 protein levels in **E** (*n* = 3). Data are presented as the mean ± SEM. ***P < 0.001, by 1-way ANOVA with Tukey’s post hoc test for multiple comparisons.

To investigate the role of WDR26 in the pathogenesis of Skraban-Deardorff syndrome, we established conventional KO mice. For these KO mice, exons 2–3 were selected as the target region of the *Wdr26* gene (Figure 1A). These exons span a 157 bp coding region (28.7% of the total coding sequence) and are part of a 3.9 kb KO region (Supplemental Figure 1A; supplemental material available online with this article; https://doi.org/10.1172/JCI195537DS1). We performed PCR to genotype embryos, as shown in Figure 1B. Notably, no *Wdr26^–/–^* pups were born during the mating trials. At E17.5, *Wdr26^–/–^* embryos displayed embryonic lethality, characterized by reduced body size and abnormally yellowed skin (Figure 1C). In contrast, we observed no discernible phenotypic differences between WT (*Wdr26^+/+^*) and *Wdr26^+/–^* mice (Figure 1D). There was no significant difference in body weights between the 2 groups (Supplemental Figure 1B). To further validate the KO effect on WDR26 protein expression, we analyzed WDR26 protein levels using an anti-WDR26 antibody in embryonic brain tissue from *Wdr26^+/+^*, *Wdr26^+/–^*, and *Wdr26^–/–^* embryos. The results confirmed the absence of WDR26 protein expression in *Wdr26^–/–^* embryos, whereas *Wdr26^+/–^* embryos showed an approximately 40% reduction in protein levels compared with *Wdr26^+/+^* embryos (Figure 1, E and F).

### Wdr26^+/–^ mice exhibit cognitive deficits.

**Figure 2:**
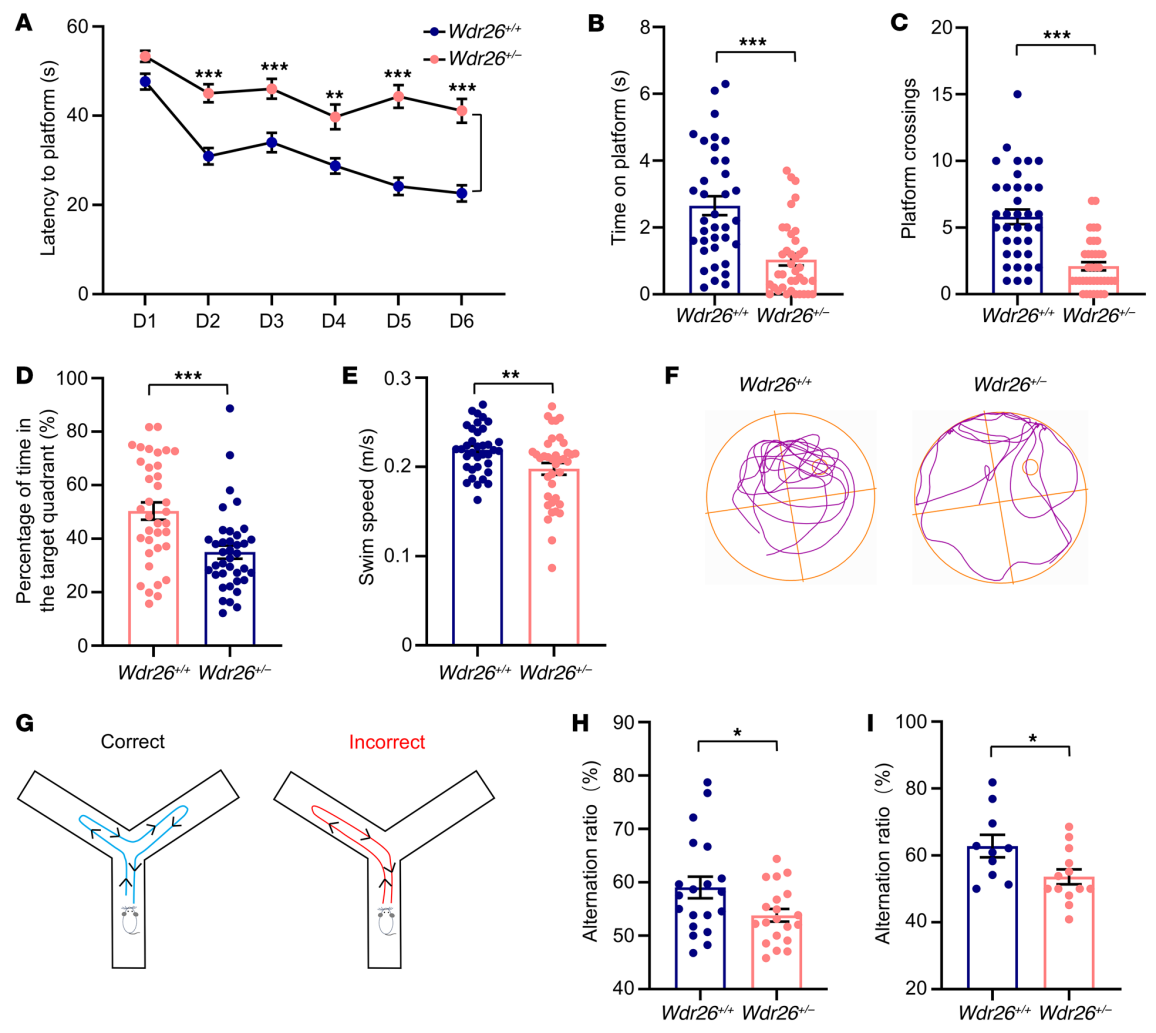
*Wdr26^+/–^* mice exhibit impairments in learning and memory. (**A**–**F**) Two-month-old *Wdr26^+/+^* and *Wdr26^+/–^* mice were tested in the MWM (*n* = 36–38). Escape to the platform latency (**A**), time spent on the platform (**B**), platform crossings (**C**), percentage of time in the target quadrant (**D**), swim speed (**E**), and representative trajectories (**F**). (**G**–**I**) Two-month-old *Wdr26^+/+^* and *Wdr26^+/–^* mice were tested in the Y-maze. Schematic of a typical Y-maze (**G**). Alternation ratios for male mice (**H**) (*n* = 20) and female mice (**I**) (*n* = 10–13). Data are presented as the mean ± SEM. *P < 0.05, **P < 0.01, and ***P < 0.001, by 2-way ANOVA with Bonferroni’s post hoc test for multiple comparisons (**A**) and unpaired, 2-tailed Student’s *t* test (**B**–**E**, **H**, and **I**).

Since Skraban-Deardorff syndrome is a neurodevelopmental disorder primarily characterized by intellectual disability (1), we assessed whether *Wdr26^+/–^* mice exhibit cognitive deficits using the Morris water maze (MWM) test of learning and memory. *Wdr26^+/–^* mice demonstrated much longer escape latencies compared with *Wdr26^+/+^* mice during the MWM training phase (Figure 2A). We found no sex difference. Both *Wdr26^+/–^* male (Supplemental Figure 2A) and female (Supplemental Figure 2B) mice exhibited longer escape latencies. In the testing phase, *Wdr26^+/–^* mice spent less time on the platform (Figure 2B) and made fewer platform crossings (Figure 2C) than did their *Wdr26^+/+^* counterparts, in both male (Supplemental Figure 2, C and D) and female (Supplemental Figure 2, E and F) mice. Additionally, *Wdr26^+/–^* mice spent a smaller percentage of time in the target quadrant (Figure 2D). In terms of sex differences, *Wdr26^+/–^* male mice spent a significantly reduced percentage of time in the target quadrant (Supplemental Figure 2G), whereas a decreasing trend was observed among female mice, although it did not reach statistical significance (Supplemental Figure 2H). The swimming speed of *Wdr26^+/–^* mice is shown in Figure 2E, with no significant difference observed between male *Wdr26^+/–^* and *Wdr26^+/+^* mice (Supplemental Figure 2I), whereas female *Wdr26^+/–^* mice exhibited a slight reduction in swim speed (Supplemental Figure 2J). *Wdr26^+/–^* mice exhibited altered locomotor trajectories (Figure 2F). We evaluated spatial working memory using the Y-maze, where correct entries into different arms were defined as an alternation and incorrect entries as a nonalternation (Figure 2G). Both male (Figure 2H) and female (Figure 2I) *Wdr26^+/–^* mice exhibited significantly lower alternation rates compared with *Wdr26^+/+^* mice, indicating impaired spatial working memory.

### Wdr26^+/–^ mice exhibit social deficits and increased seizure susceptibility.

**Figure 3:**
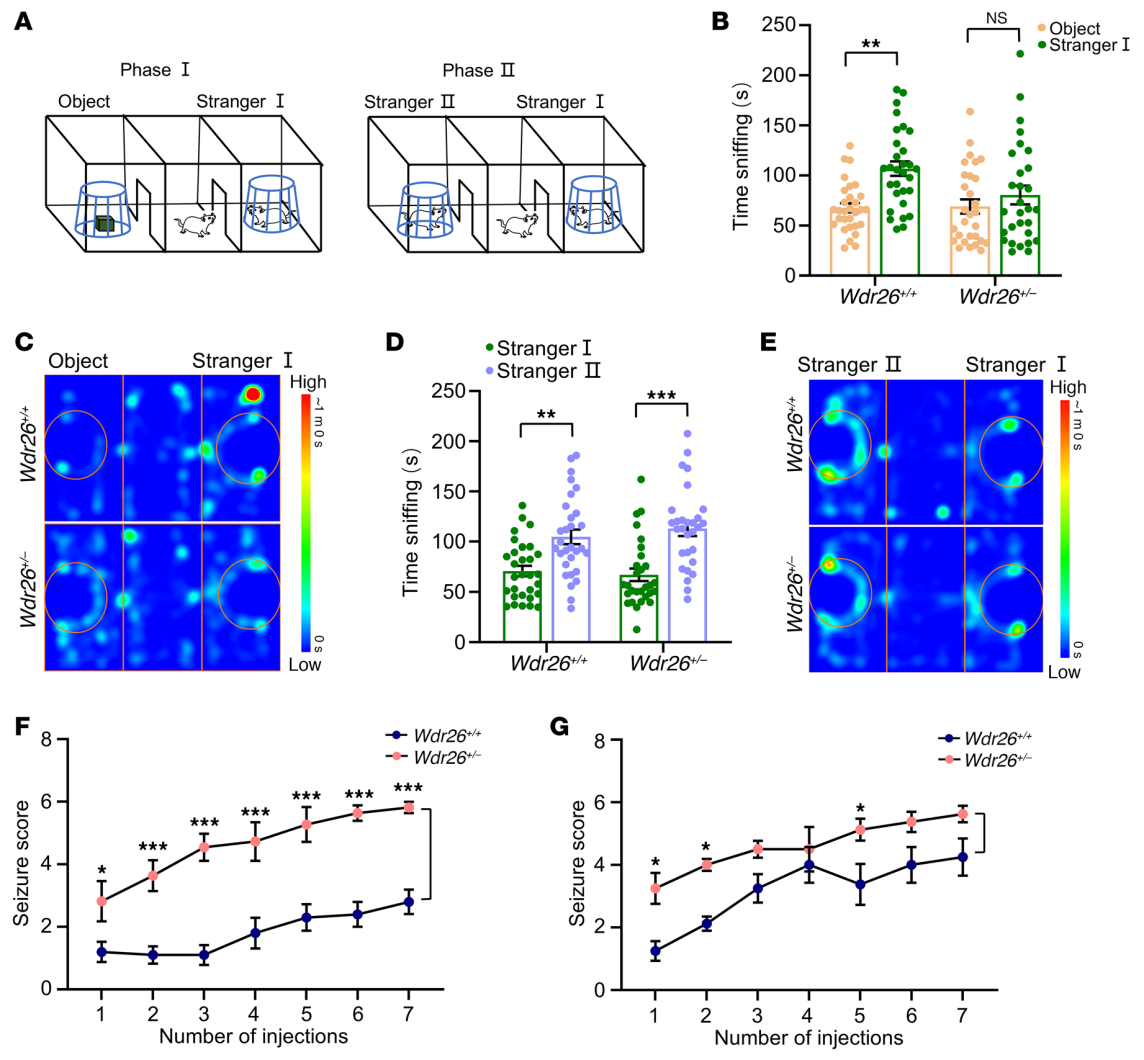
*Wdr26^+/–^* mice exhibit sociability deficits and high susceptibility to epilepsy. (**A**) Schematic of a typical 3-chamber socialization test, conducted in 2 phases. (**B**) Quantification of the investigative behaviors of 2-month-old *Wdr26^+/+^* and *Wdr26^+/–^* mice during phase I of the 3-chamber socialization test (*n* = 29–30 mice). (**C**) Representative trajectories of mice during phase I of the 3-chamber socialization test. (**D**) Quantification of the investigative behaviors of 2-month-old *Wdr26^+/+^* and *Wdr26^+/–^* mice during phase II of the 3-chamber socialization test (*n* = 29–30 mice). (**E**) Representative trajectories of mice during phase II of the 3-chamber socialization test. (**F** and **G**) Seizure scores for *Wdr26^+/+^* and *Wdr26^+/–^* mice treated with PTZ were assessed. *n* = 10–11 male mice (**F**); *n* = 8 female mice (**G**). Data are presented as the mean ± SEM. *P < 0.05, **P < 0.01, and ***P < 0.001, by 2-way ANOVA, with Tukey’s post hoc test for multiple comparisons (**B** and **D**) and Holm-Šidák’s post hoc test (**F** and **G**).

Because patients with Skraban-Deardorff syndrome often exhibit autistic behaviors and seizures (1, 5–11), we conducted the 3-chamber socialization test on 8-week-old *Wdr26^+/+^* and *Wdr26^+/–^* mice to assess social behaviors. The experimental design of the 3-chamber test is illustrated in Figure 3A. *Wdr26^+/+^* mice spent significantly more time on social interactions than on object sniffing in phase I, whereas *Wdr26^+/–^* mice failed to show this distinction (Figure 3B), and this was applicable to both males (Supplemental Figure 3A) and females (Supplemental Figure 3B). The representative trajectories of mice are shown in Figure 3C. During phase II of the test, both *Wdr26^+/–^* and *Wdr26^+/+^* mice showed increased social interactions with stranger II compared with stranger I, indicating a preserved social novelty preference among *Wdr26^+/–^* mice (Figure 3, D and E). We observed a similar pattern in male *Wdr26^+/–^* mice (Supplemental Figure 3C) and female *Wdr26^+/–^* mice (Supplemental Figure 3D).

Further, to evaluate seizure susceptibility, *Wdr26^+/–^* mice and *Wdr26^+/+^* mice were treated with pentylenetetrazol (PTZ), a GABA-A receptor antagonist, via intraperitoneal injection at a dose of 35 mg/kg every other day to induce seizures, and the seizure scores were recorded. Both male (Figure 3F) and female (Figure 3G) *Wdr26^+/–^* mice had significantly higher seizure scores than did *Wdr26^+/+^* mice following PTZ administration.

### Wdr26^+/–^ mice display motor dysfunction.

**Figure 4:**
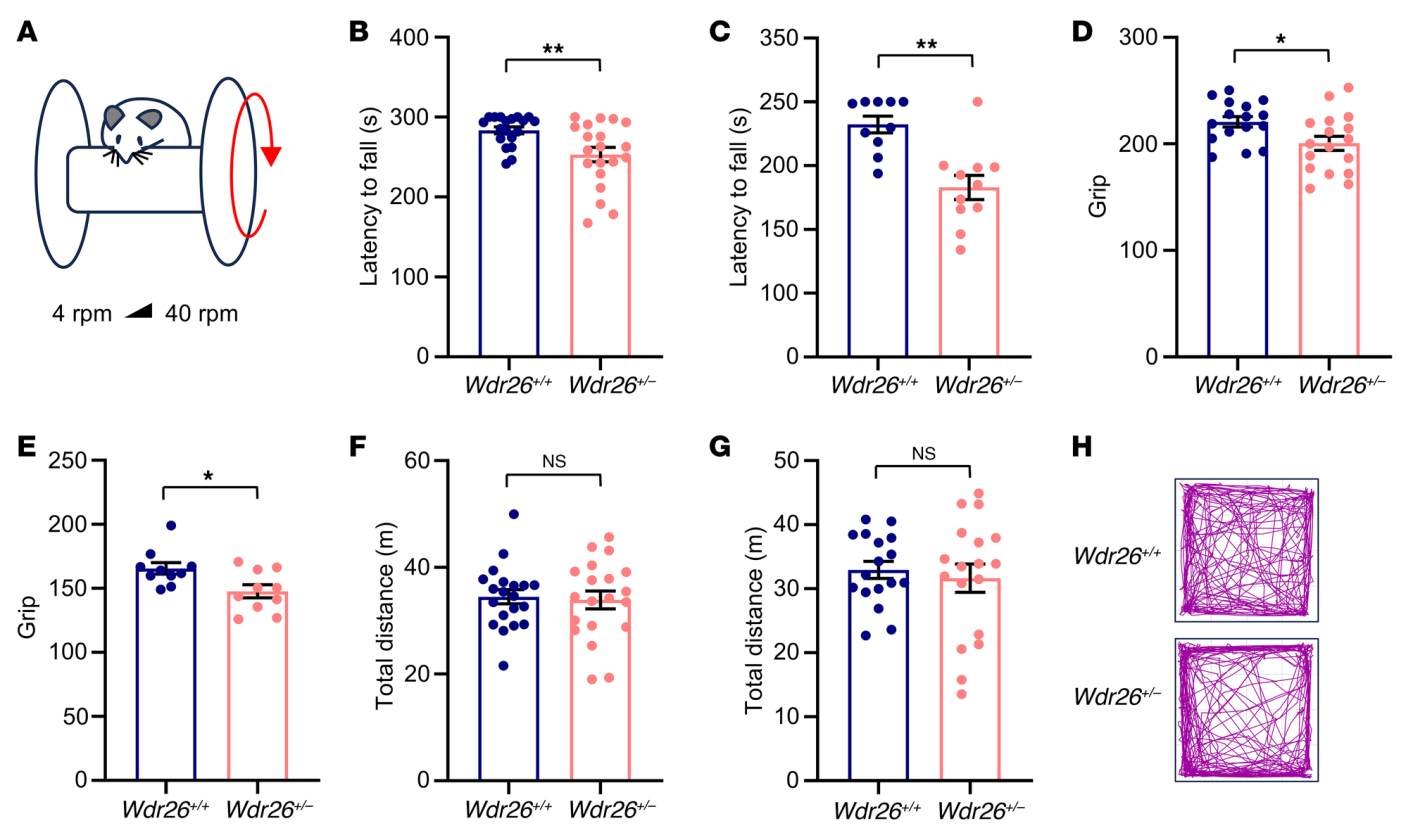
The motor function of *Wdr26^+/–^* mice is impaired. (**A**–**C**) *Wdr26^+/+^* and *Wdr26^+/–^* mice were subjected to the rotarod test. Schematic of the rotarod test (**A**). Latency to fall results for male (**B**) and female (**C**) mice. *n* = 19–20 male mice (**B**); *n* = 10–11 female mice (**C**). (**D** and **E**) The grip strength of *Wdr26^+/+^* and *Wdr26^+/–^* male mice (**D**) and female mice (**E**) was measured. *n* = 16–17 male mice (**D**); *n* = 10 female mice (**E**). (**F**–**H**). *Wdr26^+/+^* and *Wdr26^+/–^* mice were subjected to the open-field test. Total distance for male *Wdr26^+/+^* and *Wdr26^+/–^* mice (**F**), total distance for female *Wdr26^+/+^* and *Wdr26^+/–^* mice (**G**). *n* = 20 male mice (**F**); *n* = 17–18 female mice (**G**). Representative trajectories (**H**). Data are presented as the mean ± SEM. *P < 0.05 and **P < 0.01, by unpaired, 2-tailed Student’s *t* test.

Given the characteristic motor impairments including ataxic-like gait in patients with Skraban-Deardorff syndrome (1, 5–11), we evaluated motor function in *Wdr26^+/+^* and *Wdr26^+/–^* mice using a series of behavioral tests. The rotarod test was conducted to evaluate motor coordination, balance, and endurance, with an accelerated speed ranging from 4 rpm to 40 rpm within a 5-minute period (Figure 4A). After a 2-day training period, *Wdr26^+/–^* mice demonstrated a significantly shorter latency to fall than did *Wdr26^+/+^* mice, a trend observed in both males (Figure 4B) and females (Figure 4C). Furthermore, grip strength measurements revealed a slight but consistent reduction in *Wdr26^+/–^* mice (Figure 4, D and E), further supporting the presence of motor dysfunction. In the open-field test, no differences in the total distance traveled were observed between *Wdr26^+/–^* and *Wdr26^+/+^* male mice (Figure 4F) and female mice (Figure 4G). However, *Wdr26^+/–^* mice spent significantly less time in the center of the arena (Supplemental Figure 4, A, B, and H), suggesting increased anxiety-like behaviors.

### Wdr26 insufficiency causes developmental abnormalities.

**Figure 5:**
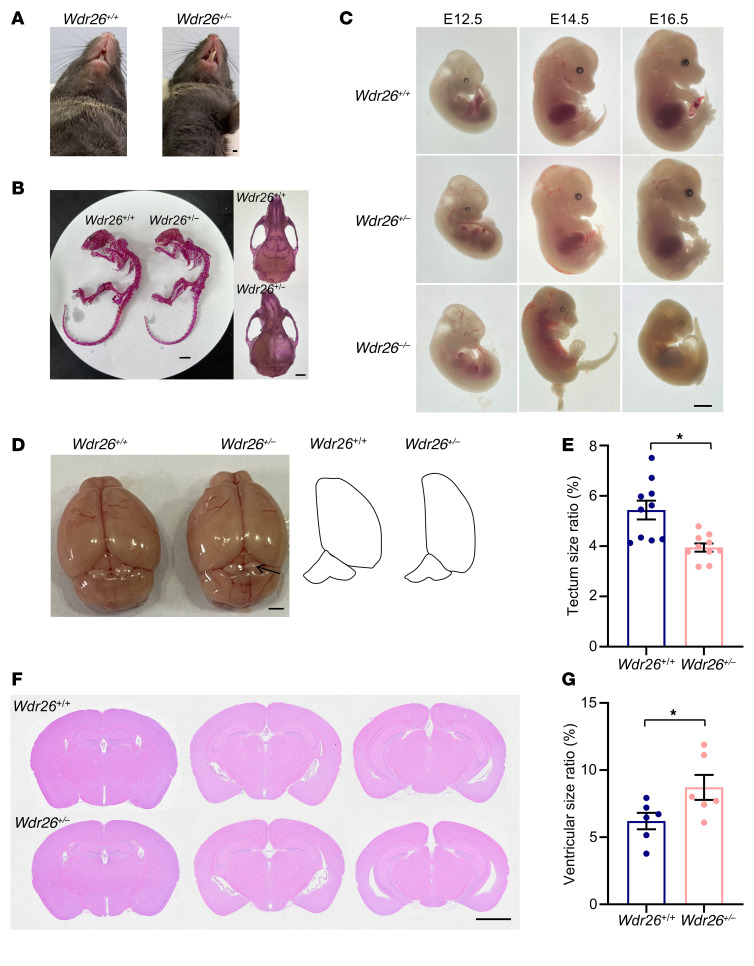
*Wdr26^+/–^* mice show developmental abnormalities, with a small proportion exhibiting dental malformations and cranial abnormalities. (**A** and **B**) Images of *Wdr26^+/+^* and *Wdr26^+/–^* mice at 6–8 weeks of age. (**A**) *Wdr26^+/–^* mice exhibited tooth deformities (8 of 132). (**B**) Skeletal staining shows cranial misalignment in *Wdr26^+/–^* mice (8 of 132). (**C**) *Wdr26^+/+^*, *Wdr26^+/–^*, and *Wdr26^–/–^* embryos at E12.5, E14.5, and E16.5. Images are representative of 3 independent experiments (**A**–**C**). (**D**) Images of brains from 2-month-old *Wdr26^+/+^* and *Wdr26^+/–^* mice and a schematic outline of the corresponding right cerebral cortex and tectum. The arrow indicates the tectum. (**E**) Quantitative analysis of the tectum size in **D** (*n* = 10). Tectum size ratio (percentage) = (tectum area/total brain area) × 100%. (**F**) H&E staining of brain sections in *Wdr26^+/+^* and *Wdr26*^+/–^ mice. (**G**) Quantitative analysis of ventricular sizes in **F** (*n* = 6). The ventricular size ratio refers to the average percentage of the ventricular area relative to the total brain area, calculated as the average of area ratios in the 3 sections (**F**). Scale bars: 2 mm (**A**), 1 cm (**B**, left), 2 mm (**B**, right), 2 mm (**C**), 1 mm (**D**), and 2 mm (**F**).

Facial deformities, such as a prominent maxilla and widely spaced teeth, commonly observed in patients with Skraban-Deardorff syndrome (1, 5–11), were present in approximately 6% of *Wdr26^+/–^* mice, which had abnormal tooth growth (Figure 5A) and cranial misalignment (Figure 5B). Because developmental abnormalities were observed in *Wdr26^–/–^* embryos at E17.5 (Figure 1C), we further examined embryos at earlier developmental stages (E12.5, E14.5, and E16.5). Our results revealed no significant differences between *Wdr26^+/+^* and *Wdr26^+/–^* embryos; however, *Wdr26^–/–^* embryos at E14.5 were remarkably smaller than their *Wdr26^+/+^* and *Wdr26^+/–^* counterparts, and this size disparity persisted at E16.5 and was accompanied by pronounced skin yellowing (Figure 5C). In adult mice, *Wdr26^+/–^* mice showed a smaller tectum compared with *Wdr26^+/+^* mice (Figure 5, D and E). Additionally, an enlarged ventricular area was observed in the brains of *Wdr26^+/–^* mice (Figure 5, F and G), similar to that seen in patients with Skraban-Deardorff syndrome (1, 5–11). Collectively, these findings demonstrate that WDR26 deficiency severely disrupted embryonic development, while reduced WDR26 expression was linked to abnormalities of the craniofacial, tectum, and ventricular areas.

### WDR26 insufficiency alters protein expression patterns.

**Figure 6:**
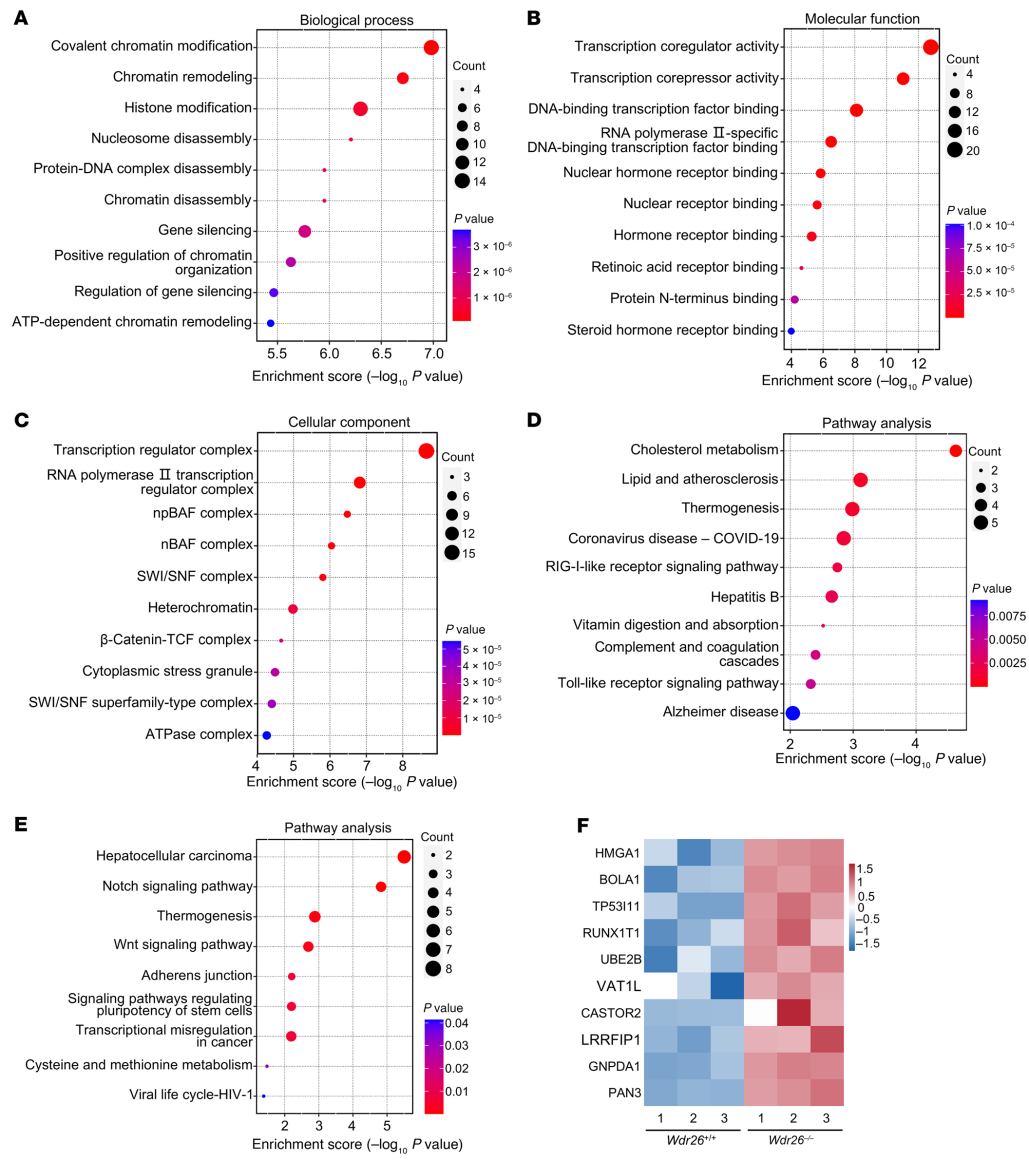
Loss of *Wdr26* induces aberrant protein expression patterns. (**A**–**C**) GO analysis of the upregulated proteins. Biological process (**A**), molecular function (**B**), and cellular component (**C**). (**D**) KEGG analysis of the downregulated proteins. (**E**) KEGG analysis of the upregulated proteins. (**F**) Heatmap showing the top 10 upregulated proteins.

To elucidate the molecular mechanisms underlying the behavioral phenotypes observed in *Wdr26^+/–^* mice, we performed proteomic mass spectrometry analysis of brain tissue from *Wdr26^+/+^* and *Wdr26^–/–^* embryos (E12.5). The analysis identified a total of 393 differentially expressed proteins (DEPs), including 231 upregulated and 162 downregulated proteins (Supplemental Figure 5A). Gene Ontology (GO) analysis of downregulated proteins revealed their involvement in lipid metabolism and mitochondrial function (Supplemental Figure 5B), while upregulated proteins were associated with chromatin and histone modifications, as well as gene silencing in biological processes (Figure 6A). Molecular functions related to transcription regulation, such as transcription coregulator activity, transcription corepressor activity, and DNA-binding transcription factor binding, were significantly enriched (Figure 6B). Cellular component analysis highlighted processes linked to transcription regulation, including the transcription regulator complex and the RNA polymerase II transcription regulator complex (Figure 6C). Kyoto Encyclopedia of Genes and Genomes (KEGG) analysis further demonstrated that downregulated proteins were enriched in pathways related to cholesterol metabolism, lipid and atherosclerosis, and Alzheimer’s disease (Figure 6D). In contrast, upregulated proteins were associated with the Notch signaling pathway and the Wnt signaling pathway (Figure 6E), which are required for embryonic development (26–29). A comprehensive analysis of all DEPs indicated connections to several neurodegenerative diseases, including Alzheimer’s disease, amyotrophic lateral sclerosis, Huntington’s disease, and Parkinson’s disease (Supplemental Figure 6A). To identify key proteins, we focused on the top 10 upregulated proteins, which included HMGA1, BOLA1, TP53I11, RUNX1T1, UBE2B, VAT1L, CASTOR2, LRRFIP1, GNPDA1, and PAN3 (Figure 6F). Among these proteins, RUNX1T1, implicated in intellectual disability, plays a critical role in brain development and neuronal differentiation (30–32).

### Wdr26 haploinsufficiency increases RUNX1T1 protein levels by impairing its ubiquitination.

**Figure 7:**
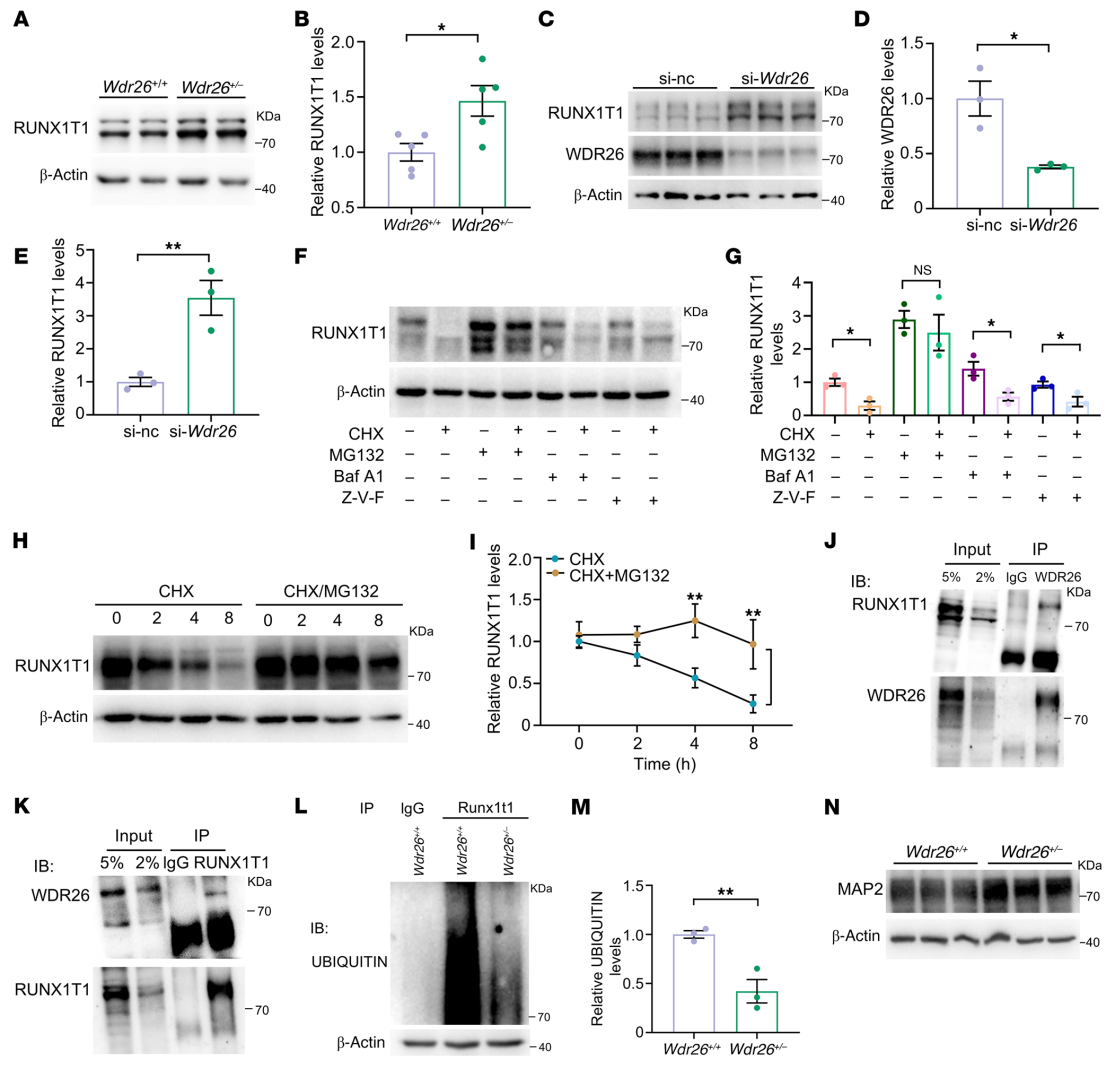
WDR26 interacts with RUNX1T1 to enhance its ubiquitination, thereby regulating protein levels. (**A**) mPFC tissues from *Wdr26^+/+^* and *Wdr26^+/–^* mice were collected for Western blotting. (**B**) Quantitative analysis of RUNX1T1 protein levels (*n* = 5) from **A**. (**C**) N2a cells transfected with si-*Wdr26* for 36 hours were tested for RUNX1T1 and WDR26 protein levels by Western blotting. (**D**) Quantitative analysis of WDR26 protein levels from **C** (*n* = 3). (**E**) Quantitative analysis of RUNX1T1 protein levels from **C** (*n* = 3). (**F**) N2a cells were treated with CHX, MG132, bafilomycin A1 (Baf A1), and Z-VAD-FMK (Z-V-F) for 12 hours, and RUNX1T1 protein levels were measured by Western blotting. (**G**) Quantitative analysis of RUNX1T1 protein levels from **F** (*n* = 3). (**H**) N2a cells were treated with CHX and MG132 for 2, 4, and 8 hours and RUNX1T1 protein levels were measured by Western blotting. (**I**) Quantitative analysis of RUNX1T1 protein levels from **H** (*n* = 3). (**J** and **K**). Immunoprecipitation analysis of WDR26 and RUNX1T1 interactions in the brains of *Wdr26^+/+^* mice. Data are representative of 3 independent experiments. (**L**) Immunoprecipitation analysis of ubiquitination of RUNX1T1 in the brains of *Wdr26^+/+^* and *Wdr26^+/–^* mouse embryos. (**M**) Quantitative analysis of ubiquitination levels from **L** (*n* = 3). (**N**) MAP2 levels in the mPFC of *Wdr26^+/+^* and *Wdr26^+/–^* mice at P0.5 were measured by Western blotting (*n* = 3). Data are presented as the mean ± SEM. *P < 0.05 and **P < 0.01, by unpaired, 2-tailed Student’s *t* test (**B**, **D**, **E**, **G**, and **M**) and 2-way ANOVA with Tukey’s post hoc test for multiple comparisons (**I**).

To investigate the regulatory effect of WDR26 on RUNX1T1, we analyzed the protein levels of RUNX1T1 in *Wdr26^+/+^* and *Wdr26^+/–^* mice using Western blotting (Figure 7A). The results showed that RUNX1T1 levels were significantly elevated in *Wdr26^+/–^* mice compared with *Wdr26^+/+^* mice (Figure 7B). Furthermore, we transfected N2a cells with *Wdr26* siRNA and found that WDR26 protein levels decreased drastically (Figure 7, C and D). However, at the same time, the levels of RUNX1T1 increased, which was consistent with the results observed in *Wdr26^+/–^* mice (Figure 7, C and E).

Because WDR26 functions as a core subunit of an E3 ubiquitin ligase complex to promote protein degradation (19, 33), we examined the main degradation pathways of RUNX1T1, including the ubiquitin-proteasome pathway, the autophagic-lysosomal pathway, and the caspase-dependent apoptotic pathway. We treated N2a cells with the protein synthesis inhibitor cycloheximide (CHX), the proteasome inhibitor MG132, the autophagy inhibitor bafilomycin A1, and the apoptosis inhibitor Z-VAD-FMK. Treatment with CHX alone led to a rapid reduction in RUNX1T1 levels. Cotreatment with bafilomycin A1 or Z-VAD-FMK did not restore RUNX1T1 levels. However, cotreatment with MG132 significantly elevated RUNX1T1 levels compared with treatment with CHX alone (Figure 7, F and G, and Supplemental Figure 7, A and B). Treatment with MG132 alone also effectively increased the levels of RUNX1T1 (Supplemental Figure 7, C and D). Subsequently, we examined the effect of MG132 on RUNX1T1 protein levels under CHX treatment at 2, 4, and 8 hours (Figure 7H). Western blotting results showed that CHX continuously inhibited the protein levels of RUNX1T1 in a time-dependent manner, while cotreatment with MG132 partially reversed this inhibition, and a significant difference was observed between 4 and 8 hours (Figure 7I). These findings indicate that RUNX1T1 is primarily degraded via the ubiquitin-proteasome pathway rather than the autophagy/lysosomal or caspase-dependent apoptotic pathways.

Next, to determine whether WDR26 interacts with RUNX1T1 to facilitate its ubiquitination, we performed co-immunoprecipitation experiments using agarose beads preincubated with an anti-WDR26 antibody. The results showed that RUNX1T1 was present in the eluate, whereas no RUNX1T1 was detected in the control samples incubated with anti-IgG (Figure 7J). Consistent with this result, Western blotting with an anti-RUNX1T1 antibody confirmed the presence of WDR26 in the co-immunoprecipitation (Figure 7K). Moreover, a significant decrease in the ubiquitination of RUNX1T1 was observed in *Wdr26^+/–^* embryos compared with *Wdr26^+/+^* embryos (Figure 7, L and M), indicating that *Wdr26* haploinsufficiency downregulated the ubiquitination of RUNX1T1.

Previous studies have shown that RUNX1T1 plays a role in the development and maintenance of microtubule-associated protein 2–positive (MAP2^+^) neurons (32, 34). In this study, we explored whether the upregulation of RUNX1T1 induced by WDR26 deficiency affects the expression of MAP2. Western blotting analysis revealed a significant increase in MAP2 levels in the medial prefrontal cortex (mPFC) of *Wdr26^+/–^* mice (Figure 7N and Supplemental Figure 7E), suggesting that the reduction of WDR26 may play a critical role in the pathogenesis of Skraban-Deardorff syndrome by increasing the protein stability of RUNX1T1 and neuronal maturation.

### Neonatal Runx1t1 knockdown rescues cognitive deficits in Wdr26^+/–^ mice.

**Figure 8:**
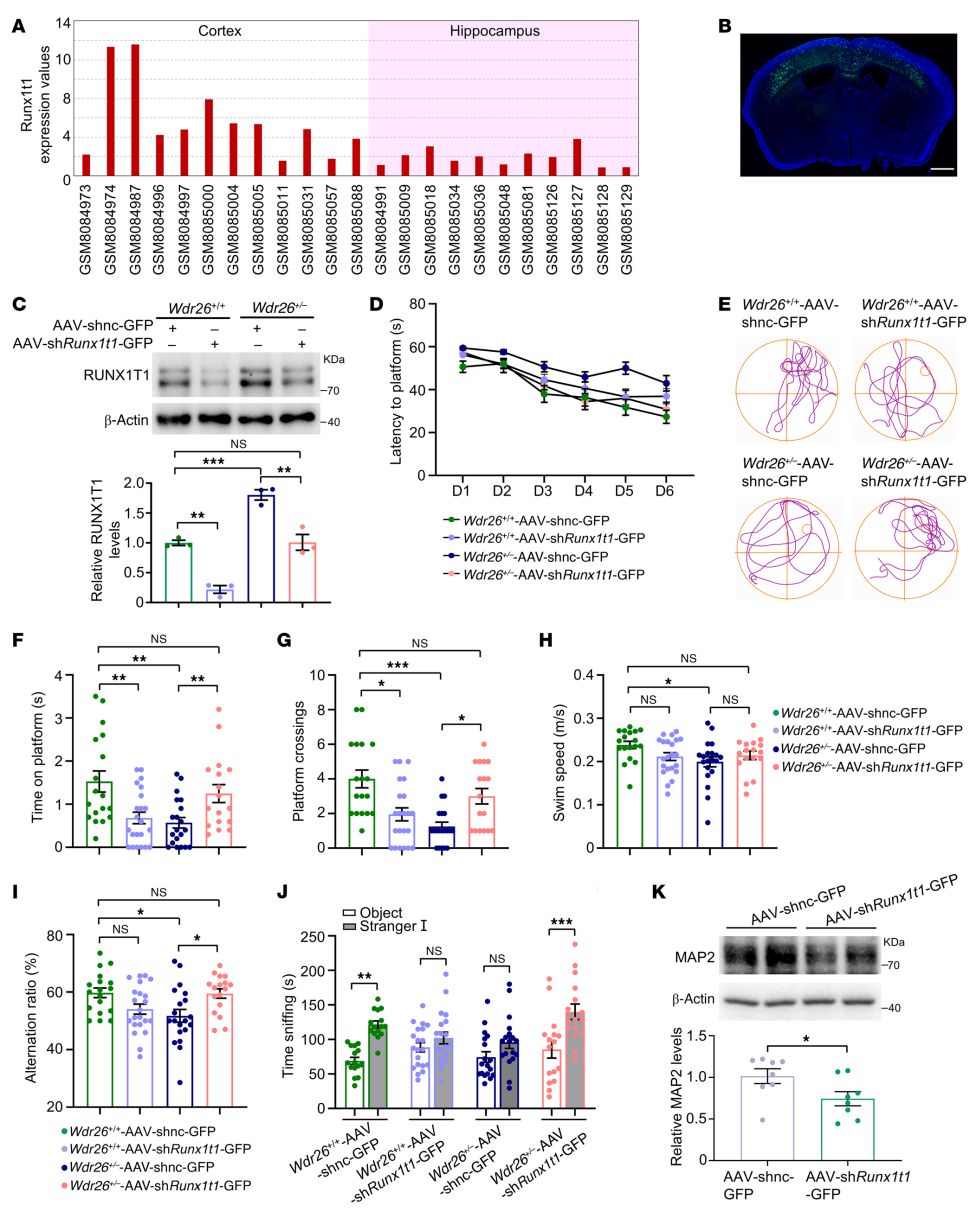
AAV-sh*Runx1t1-*GFP treatment rescues impaired behaviors in *Wdr26^+/–^* mice. (**A**) *Runx1t1* mRNA levels in the cortex and hippocampus of the human control group (*P* < 0.05). (**B**) AAV-sh*Runx1t1-*GFP was injected into the cortex of *Wdr26^+/–^* mice. Green fluorescence was derived from GFP, which is coexpressed with sh*Runx1t1*. Scale bar: 2 mm. Data are representative of 3 independent experiments. (**C**) RUNX1T1 levels were tested in the cortex of *Wdr26^+/+^* and *Wdr26^+/–^* mice by Western blotting. (**D**–**H**). *Wdr26^+/+^* and *Wdr26^+/–^* mice injected with AAV-shnc*-*GFP or AAV-sh*Runx1t1-*GFP were tested in the MWM. Escape latency (**D**), representative trajectories (**E**), time spent on the platform (**F**), platform crossings (**G**), and swim speed (**H**) (*n* =17–22). (**I**) *Wdr26^+/+^* and *Wdr26^+/–^* mice injected with AAV-shnc-GFP or AAV-sh*Runx1t1-*GFP were tested in the Y-maze (*n* = 17–22). (**J**) Quantification of investigative behaviors of *Wdr26^+/+^* and *Wdr26^+/–^* mice injected with AAV-shnc-GFP or AAV-sh*Runx1t1-*GFP during phase I of the 3-chamber socialization test (*n* =15–20). (**K**) MAP2 levels in *Wdr26^+/–^* mice injected with AAV-shnc-GFP or AAV-sh*Runx1t1* were measured by Western blotting (*n* = 8). Data are presented as the mean ± SEM. *P < 0.05, **P < 0.01, and ***P < 0.001, by 1-way ANOVA with Tukey’s post hoc test for multiple comparisons (**C**, **F**, and **I**) or Dunn’s post hoc test (**G** and **H**); 2-way ANOVA with Tukey’s post hoc test (**J**); and unpaired, 2-tailed Student’s *t* test (**K**).

Notably, *RUNX1T1* levels were markedly higher in the cortex compared with the hippocampus in the human control group through reanalysis of data from the Gene Expression Omnibus (GEO) database (GSE256068, Figure 8A). On the basis of these observations, we targeted *Runx1t1* in the cortex of neonatal *Wdr26^+/+^* and *Wdr26^+/–^* mice (P0.5) by microinjecting adeno-associated virus–*shRunx1t1*-GFP (AAV-sh*Runx1t1*-GFP) or AAV-shnc-GFP. After 21 days, adenovirus infiltration was confirmed (Figure 8B). Western blot analysis revealed a significant reduction in RUNX1T1 protein levels in mice treated with AAV-sh*Runx1t1*-GFP compared with those treated with the control vector AAV-shnc-GFP (Figure 8C). Furthermore, behavioral tests were performed in AAV-sh*Runx1t1*-GFP–treated mice at 6 weeks after injection. In the MWM training, in comparison with AAV-shnc-GFP, treatment with AAV-sh*Runx1t1*-GFP shortened the latency to find the platform for *Wdr26^+/–^* mice (Figure 8D). Trajectories from the MWM testing are illustrated in Figure 8E. *Wdr26^+/–^* mice treated with AAV-sh*Runx1t1*-GFP also spent more time on the platform (Figure 8F) and did more platform crossings than the AAV-shnc-GFP–treated controls (Figure 8G), despite similar swimming speeds (Figure 8H). In the Y-maze test, *Wdr26^+/–^* mice receiving AAV-sh*Runx1t1*-GFP had a higher spontaneous alternation ratio than did AAV-shnc-GFP–treated controls (Figure 8I). Additionally, *Runx1t1* knockdown significantly enhanced social behaviors in the 3-chamber socialization test among *Wdr26^+/–^* mice (Figure 8J). Moreover, we observed a significant decrease in the MAP2 levels in *Wdr26^+/–^* mice treated with AAV-sh*Runx1t1*-GFP compared with those treated with the control vector AAV-shnc-GFP (Figure 8K).

### Risperidone ameliorates cognitive impairment in Wdr26^+/–^ mice by elevating WDR26 levels.

**Figure 9:**
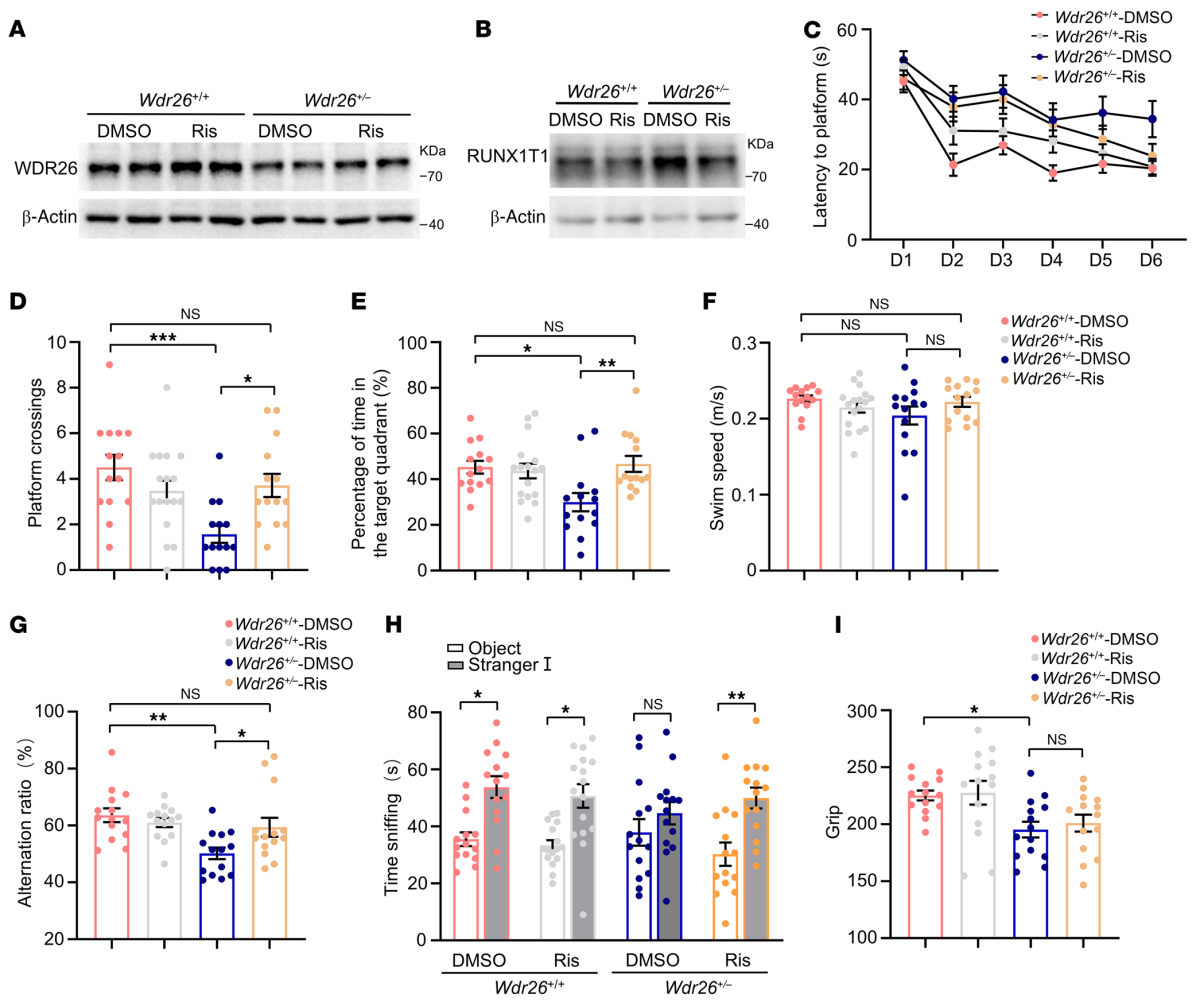
Risperidone upregulates WDR26 and ameliorates behavioral impairments in *Wdr26^+/–^* mice. (**A** and **B**) Following 21 days of risperidone treatment, WDR26 (**A**) and RUNX1T1 (**B**) protein levels in the mPFC of *Wdr26^+/+^* and *Wdr26^+/–^* mice were measured by Western blotting. (**C**–**F**) *Wdr26^+/+^* and *Wdr26^+/–^* mice treated with risperidone (Ris) were tested in the MWM. Escape latency (**C**), platform crossings (**D**), percentage of time in the target quadrant (**E**), and swim speed (**F**) (*n* = 14–17). (**G**) *Wdr26^+/+^* and *Wdr26^+/–^* mice treated with risperidone were tested in the Y-maze (*n* = 14–17). (**H**) Quantification of investigative behaviors of *Wdr26^+/+^* and *Wdr26^+/–^* mice treated with risperidone during phase I of the 3-chamber socialization test (*n* = 14–17). (**I**) Grip strength of *Wdr26^+/+^* and *Wdr26^+/–^* mice treated with risperidone was tested (*n* = 14–17). Data are presented as the mean ± SEM. *P < 0.05, **P < 0.01, and ***P < 0.001, by 1-way ANOVA with Tukey’s post hoc test for multiple comparisons (**D**–**G** and **I**) and 2-way ANOVA with Tukey’s post hoc test for multiple comparisons (**H**).

To address the behavioral deficits observed in *Wdr26^+/–^* mice and to identify compounds that could upregulate WDR26 levels, we initially performed an in vitro screen of several small-molecule libraries using HT22 cells, with WDR26 protein levels assessed by Western blotting. On the basis of this screen, compounds reported to modulate or to be implicated in neuronal activity were advanced to in vivo testing in mice. Among these, risperidone significantly upregulated WDR26 levels in HT22 cells (Supplemental Figure 8, A and B). Based on this finding, we administered risperidone (1.5 mg/kg) intraperitoneally to *Wdr26^+/+^* and *Wdr26^+/–^* mice. In *Wdr26^+/–^* mice, risperidone treatment resulted in increased WDR26 levels (Figure 9A and Supplemental Figure 8C) and decreased RUNX1T1 levels compared with DMSO treatment (Figure 9B and Supplemental Figure 8D).

To evaluate whether risperidone alleviates behavioral deficits, we assessed learning, memory, social behavior, and grip strength in the treated mice. Following a 21-day daily risperidone treatment, *Wdr26^+/–^* mice exhibited a shorter escape latency during the MWM training phase compared with those treated with DMSO (Figure 9C). In the testing phase, risperidone-treated *Wdr26^+/–^* mice had a greater number of platform crossings (Figure 9D) and spent a higher percentage of time in the target quadrant (Figure 9E) than the DMSO-treated group. No significant differences in swimming speed were observed between the groups (Figure 9F). In the Y-maze test, risperidone treatment significantly improved the spontaneous alternation ratio compared with DMSO treatment in *Wdr26^+/–^* mice (Figure 9G). Additionally, risperidone enhanced social interaction duration in the 3-chamber test for *Wdr26^+/–^* mice (Figure 9H). However, grip strength remained unchanged between the risperidone- and DMSO-treated groups (Figure 9I).

## Discussion

Skraban-Deardorff syndrome, a rare neurodevelopmental disorder caused by *WDR26* haploinsufficiency, presents with intellectual disability, seizures, autistic-like behaviors, and craniofacial anomalies (1, 5–11). While prior studies established *WDR26* loss-of-function variants and microdeletions as the genetic basis of the syndrome (1, 5–11, 25), the molecular mechanisms linking disrupted E3 ubiquitin ligase activity to neurodevelopmental deficits remained unresolved. Our study bridges this critical gap by demonstrating that *Wdr26* haploinsufficiency in mice recapitulates core behavioral and physiological features of the human disorder; it further identifies RUNX1T1 upregulation as a central pathogenic driver. These findings not only advance our understanding of the syndrome’s etiology but also provide a foundation for exploring targeted therapeutic strategies.

### Recapitulation of human phenotypes in Wdr26^+/–^ mice.

To date, the clinical symptoms of Skraban-Deardorff syndrome have been well documented in 30 cases (1, 5–11). Our *Wdr26^+/–^* mouse model faithfully mirrors the clinical spectrum of Skraban-Deardorff syndrome. Consistent with the 30/30 incidence of patients with intellectual disability, *Wdr26^+/–^* mice exhibited pronounced learning and memory deficits in the MWM. Eighty percent of patients (24 of 30) exhibit either clinical seizures or electroencephalographic abnormalities (1, 5–11), a phenotypic manifestation that is paralleled by increased sensitivity to pentylenetetrazol-induced seizures in *Wdr26^+/–^* mice. Similarly, social impairments resembling autistic-like behaviors in 65% of patients (15 of 23) are replicated in the reduced sociability of these mice. More than half of the patients show anteverted nares, a depressed nasal root, widely spaced teeth, and abnormal gums (1, 5–11). However, craniofacial anomalies, such as dental malformations, are less penetrant in mice (6%), likely due to anatomical differences and challenges in detecting subtle dysmorphisms in murine models. Importantly, the absence of sex bias in both patients (*n* = 13 males and *n* = 17 females) (1, 5–11) and *Wdr26^+/–^* mice underscores the utility of this model for studying sex-independent mechanisms. These phenotypic parallels validate *Wdr26^+/–^* as a robust preclinical tool for dissecting syndrome pathogenesis.

### The WDR26/RUNX1T1/MAP2 axis is involved in Skraban-Deardorff syndrome.

A key innovation of this study lies in elucidating how *Wdr26* haploinsufficiency disrupts neurodevelopmental pathways via upregulation of RUNX1T1, a transcriptional coactivator expressed in neural cells (35) that is critical for neuron subtype identity (36) and neuronal development (37) and differentiation (32, 38, 39). We demonstrate that WDR26 deficiency reduced ubiquitination and stabilized RUNX1T1 protein, leading to its aberrant accumulation in the developing brain. This finding aligns with prior work implicating WDR26 as a substrate-specific adaptor for the CTLH E3 ubiquitin ligase complex (24, 40) but extends it by identifying *Runx1t1* as a target. The link between RUNX1T1 dysregulation and Skraban-Deardorff syndrome is strengthened by overlapping clinical features. Patients with *RUNX1T1* mutations exhibit intellectual disability, developmental delays, and craniofacial defects (30, 31), mirroring the core symptoms of Skraban-Deardorff syndrome. Elevated RUNX1T1 levels in *Wdr26^–/–^* embryos correlate with transcriptional dysregulation (41, 42) and chromatin remodeling (43–45) pathways (Figure 6, A–C), potentially modulating critical brain development genes through these mechanisms. RUNX1T1’s role in neuronal maturation is underscored by the fact that its overexpression promotes MAP2^+^ neuron differentiation, whereas *Runx1t1* knockdown reduces MAP2^+^ neuron differentiation (34). Consistent with this, our data suggest that RUNX1T1 accumulation disrupts postnatal neuronal remodeling, as evidenced by elevated MAP2 levels in *Wdr26^+/–^* mice (Figure 7N). MAP2 upregulation, indicative of aberrant dendritic arborization, has been implicated in dendritic differentiation (46, 47), synaptic hyperconnectivity, and network instability (48, 49), potentially explaining the cognitive deficits and seizure propensity in both mice and patients. Notably, postnatal AAV-sh*Runx1t1-*GFP administration decreased MAP2 levels and ameliorated behavioral deficits (Figure 8). Although the majority of brain neurons do not undergo division or apoptosis postnatally, there is a direct conversion of both structural and transcriptomic types within individual cells between P7 and P28, leading to substantial alterations in the composition of structural types (50). These findings highlight the reversibility of neuronal pathology by intervention in neonatal mice and the therapeutic potential of modulating this pathway.

### Wdr26 loss disrupts chromatin and transcriptional homeostasis.

We found that *Wdr26* deficiency upregulated chromatin and transcriptional regulators (Figure 6, A–C), many of which are implicated in neurodevelopmental disorders. This upregulated set included key molecules such as SMARCB1 and SMARCA4 (core subunits of the SWI/SNF chromatin remodeling complex); DNMT1 (a DNA methyltransferase); RCOR2, NCOR1, NCOR2, SIRT6, and KDM3B (histone modifiers); as well as RUNX1T1, LDB1, ATF7IP, CTBP2, TLE1, and TLE3 (transcriptional coregulators). These coordinated changes indicate that network-wide chromatin regulation and transcriptional activity were disrupted, which is pivotal for directing the gene expression programs that underpin neural development and plasticity.

Notably, mutations in several of these upregulated proteins, including SMARCB1, SMARCA4, and FMR1, are established causes of specific neurodevelopmental syndromes (e.g., Coffin-Siris syndrome [ref. 51] and fragile X syndrome [ref. 52], respectively), highlighting the pathophysiological relevance of their dysregulation. Importantly, the clinical phenotypes of Coffin-Siris syndrome and fragile X syndrome overlap with those of Skraban-Deardorff syndrome, sharing features such as intellectual disability, epilepsy, hypotonia, and coarse facial features. This phenotypic convergence, combined with our molecular data, suggests that SMARCB1, SMARCA4, and FMR1 may act as potential downstream effectors of WDR26, and further implicates a breakdown in chromatin-transcriptional homeostasis in the pathogenesis of Skraban-Deardorff syndrome.

### Therapeutic challenges and the critical timing of intervention.

Our attempts to rescue phenotypes via *Runx1t1* knockdown reveal the delicate balance required for therapeutic intervention. While early postnatal delivery of a small dose of AAV-sh*Runx1t1-*GFP into the cortex (P0.5) corrected MAP2 levels and behavior, high-dose intracerebroventricular injections caused lethality, mirroring the death of *Runx1t1*-mutant mice soon after birth (43). Medium-dose injections induced seizures (data not shown). These outcomes underscore 2 critical points: (a) RUNX1T1 is indispensable during early neurodevelopment, and its correction must be spatially and temporally controlled to avoid overcorrection; (b) Skraban-Deardorff syndrome likely arises from cumulative effects of multiple CTLH complex substrates. Beyond RUNX1T1, WDR26 regulates pyrimidine metabolism via uridine cytidine kinase 2 (UCK2) degradation (40) and erythropoiesis via laminin B ubiquitination (24). While our focus on RUNX1T1 provides mechanistic clarity, future studies must explore whether other substrates synergistically exacerbate neurodevelopmental deficits.

Notably, in our study, AAV-sh*Runx1t1-*GFP administration induced behavioral deficits in *Wdr26^+/+^* mice, whereas the same intervention rescued phenotypic abnormalities in *Wdr26^+/–^* mice (Figure 8, D–J). This differential effect suggests the existence of an optimal dose for achieving therapeutic benefits. In *Wdr26^+/+^* mice, reducing RUNX1T1 levels below basal levels may perturb normal developmental processes, whereas in *Wdr26^+/–^* mice — in which RUNX1T1 is pathologically upregulated — the same intervention restores expression levels closer to the physiological range, thereby conferring functional benefit. These results indicate that successful therapy may depend on fine-tuning RUNX1T1 levels rather than achieving maximal knockdown, emphasizing the importance of systematic dose optimization in future translational studies.

### Translational implications and pharmacological rescue.

Despite these challenges, our pharmacological screen identified potential therapeutic agents such as risperidone. Notably, our discovery that risperidone, an atypical antipsychotic agent used to manage autism-related behaviors, upregulated WDR26 expression and rescued cognitive and social deficits in *Wdr26^+/–^* mice opens a promising therapeutic avenue. We believe this finding is particularly important, given the lack of targeted treatments for Skraban-Deardorff syndrome. Risperidone’s ability to enhance WDR26 levels suggests a feedback mechanism linking neuronal activity to CTLH complex function, although the precise molecular interplay requires further investigation. Drug repurposing of FDA-approved agents such as risperidone may expedite therapeutic development for Skraban-Deardorff syndrome.

### Limitations and future directions.

While our study establishes RUNX1T1 as a key mediator of *Wdr26*-related pathology, several questions remain unresolved. First, the potential contribution of other WDR26 substrates to the observed phenotypes requires further investigation. In this study, we prioritized the use of *Wdr26^–/–^* mouse models for proteomics profiling (Figure 6) to maximize the identification of core molecular pathways dysregulated upon *Wdr26* ablation, which enabled the robust identification of RUNX1T1 as a key mediator. Subsequent experimental validation confirmed that RUNX1T1 was also significantly upregulated in *Wdr26^+/–^* mice. Nevertheless, we acknowledge that complete loss of *Wdr26* may eliminate a critical biological signal that is only attenuated in the heterozygous setting, or that a dosage-dependent effect of WDR26 may exist, in which varying expression levels lead to distinct pathogenic outcomes. Therefore, whether additional WDR26 substrates contribute to the syndrome phenotypes warrants further exploration. Second, the mechanisms driving craniofacial anomalies — potentially involving the role of RUNX1T1 in cranial neural crest cell differentiation — are yet to be dissected. Third, our viral intervention strategies highlight the need for advanced delivery systems to achieve precise spatiotemporal control during intervention only for a period of time (specifically during neurodevelopment). Fourth, the dose-dependent effects observed in *Runx1t1* intervention studies (Figure 8) — ranging from rescue (*Wdr26^+/–^*) to impairment (*Wdr26^+/+^*) and even lethality (data not shown) — underscore the necessity of establishing precise dosing parameters in future therapeutic development. Systematic dose-response studies will be essential to determine the optimal window for *Runx1t1* modulation that maximizes therapeutic benefit while minimizing off-target effects. Finally, we also propose that screening small molecules using this mouse model holds promise for identifying potential treatments for Skraban-Deardorff syndrome.

### Conclusion.

In summary, our work delineates a WDR26/RUNX1T1/MAP2 axis as a central pathway underlying Skraban-Deardorff syndrome, connecting haploinsufficiency-induced ubiquitination defects to neuronal dysfunction. By establishing a phenocopy mouse model and identifying a druggable node (RUNX1T1), this study provides a mechanistic framework for understanding the syndrome’s neurodevelopmental sequelae. The rescue of behavioral deficits via risperidone-mediated WDR26 upregulation further highlights the potential for repurposing existing therapeutics. These findings not only advance the field of rare genetic disorders but also exemplify how dissecting ubiquitin-proteasome pathways can yield insights into brain development and disease. Future efforts to refine intervention strategies and explore multitarget approaches will be pivotal in translating these discoveries into clinical benefits for patients.

## Methods

### Sex as a biological variable.

Sex was included as a biological variable. Our study examined male and female animals, and similar findings are reported for both sexes.

### Experimental animals.

All mice were maintained in specific pathogen–free (SPF) facilities under controlled environmental conditions with ad libitum access to autoclaved food and water under a 12-hour light/12-hour dark cycle, in accordance with the Measures for the Management of Laboratory Animals at Soochow University. *Wdr26^+/–^* mice (C57BL/6J-Wdr26^em1cyagen^, serial no. KOCMP-226757-Wdr26-B6J-VA) were generated via CRISPR/Cas9-mediated gene editing by Cyagen Biosciences. The gRNA-D1 (matching forward strand of gene) was ACCGAGGTTGTTTATGCTGGGGG and gRNA-D2 (matching forward strand of gene) was ATTGAAGCCCGAGCCTTGTCTGG.

### Behavioral paradigms.

Behavioral assessments were conducted from 0800 hours to 1700 hours, following at least 30 minutes of habituation to the testing room.

### MWM test.

The MWM is a widely applied behavioral paradigm for assessing spatial learning and memory in rodents. The apparatus consisted of a circular pool maintained at 22°C–24°C. To obscure visual detection of the escape platform, the water was rendered opaque with nontoxic white tempera paint. A circular platform (diameter: 10 cm) was submerged 1 cm below the water surface, remaining invisible to the mice throughout the test. Distinct geometric shapes serving as spatial cues were positioned at fixed locations on the pool walls to aid navigation. The experiment comprised 2 phases: (a) the acquisition phase, which consisted of 6 consecutive days of training with 4 trials per day; and (b) the probe trial, which was conducted on day 7 to evaluate memory retention. During training, mice were allowed up to 60 seconds to locate the submerged platform. Mice failing to reach the platform within the allotted time were gently guided to it and permitted 20 seconds of stationary positioning to reinforce spatial memory. In the probe trial, the platform was removed, and mice freely explored the pool for 60 seconds. All movement trajectories were recorded using the ANY-maze video tracking system (Stoelting Co.), with parameters including latency to the target quadrant and path efficiency analyzed for cognitive performance.

### Y-maze.

The Y-maze is a standardized behavioral apparatus widely utilized to evaluate spatial working memory and cognitive flexibility in rodents. The maze comprises 3 identical arms (50 cm length × 10 cm width; labeled A, B, and C) radiating symmetrically from a central triangular junction, with each pair of arms forming a 120° angle. During testing, mice were placed in a randomly selected starting arm facing the distal end and permitted 5 minutes of unrestricted exploration of the maze. Behavioral parameters including the spontaneous alternation rate (percentage of consecutive entries into all 3 arms without repetition) and arm entry sequences were quantified using the ANY-maze video tracking system (Stoelting Co.).

### Rotarod test.

Motor coordination and balance in mice were evaluated using an accelerating rotarod apparatus (SA102, Sanbio). During the training phase, mice were acclimated to the rod operating at a constant speed (10 rpm) for 5 min/day over 2 consecutive days. For the formal test, the rotation speed increased progressively from 4 to 40 rpm over a 5-minute period, with each mouse undergoing 2 trials separated by 30-minute intervals. The apparatus automatically recorded the latency to fall (time until descent from the rod). Motor function was assessed by calculating the mean latency across both trials.

### Open-field test.

Spontaneous locomotion, exploratory behavior, and anxiety-related responses in mice were assessed through a 10-minute open-field test using a novel environment. The testing arena (40 cm × 40 cm × 40 cm) comprised a central zone (25% of the total area) and a peripheral zone. Mice were individually placed in the arena facing away from the central zone. Locomotor activity was automatically recorded and analyzed using the ANY-maze video tracking system (Stoelting Co.).

### Three-chamber socialization test.

The behavioral apparatus consisted of 3 interconnected chambers. During habituation, mice were acclimated to the central chamber for 5 minutes. In the testing phase I, 2 identical wire cages were positioned in the lateral chambers: one containing an inanimate novel object and the other housing an unfamiliar conspecific mouse (matched for sex and age to the subject mouse). Mice were then allowed free exploration of all chambers for 10 minutes. In the phase II, one cage contained the unfamiliar mouse from phase I, while the other cage contained a novel unfamiliar conspecific mouse (matched for sex and age to the subject mouse). Mice were then allowed to freely explore all chambers for 10 minutes. Social interaction behaviors were quantified using the ANY-maze video tracking system (Stoelting Co.).

### AAV-mediated gene knockdown.

Neonatal mice received bilateral intracerebral microinjections of AAV2/9-sh*Runx1t1*-eGFP (BrainVTA) or AAV2/9-shnc-eGFP (BrainVTA) at P0.5, with a viral titer of 1.0 E + 12 vg/mL. Each pup was administered 300 nL viral suspension via a calibrated microinjection system (10 μL syringe coupled to glass micropipette) using stereotaxic coordinates (anteroposterior [relative to bregma]: −1.0 mm, mediolateral [relative to midline]: ±1.0 mm, dorsoventral [relative to brain surface]: –0.8 mm). The *Runx1t1* short-hairpin sequence 5′-GATTGACCACAGACTAACAGA-3′ was used.

### Cell culture, transfection, and antibody reagents.

Hippocampal neuronal (HT22) cells and mouse neuro2a neuroblastoma (N2a) cells were cultured in DMEM supplemented with 10% FBS at 37°C with 5% CO_2_. HT22 cells were obtained from Procell Life Science and Technology (catalog CL-0595). N2a cells were obtained from the American Type Culture Collection (ATCC) (catalog CCL-131). *Wdr26* silencing was achieved via siRNA transfection (Lipofectamine RNAiMAX; Invitrogen, Thermo Fisher Scientific) using 50 nM duplex (5′-GACAAGCUUCAGACCUAUUUATT-3′; Sangon Biotech). Cells were harvested 36 hours after transfection for Western blotting.

The following primary antibodies were used: WDR26 (Bethyl Laboratories, A302-244A; Absea, KC-41682-T50); RUNX1T1 (Proteintech, 15494-1; Proteintech, 67086-1); ubiquitin (Santa Cruz Biotechnology, SC-8017); rabbit IgG control (Proteintech, 30000-0-AP); normal mouse IgG, (Beyotime, A7028), MAP2 (Proteintech, 17490-1-AP); and β-actin (RuiYingBio, RLM3028). The following secondary antibodies were used: HRP-conjugated rabbit IgG (Jackson ImmunoResearch, 111-035-003), HRP-conjugated mouse IgG (Jackson ImmunoResearch, 115-035-003), and a universal secondary antibody (Abmart, M21008S).

### Skeletal staining.

Skeletal staining was performed using Alcian blue and alizarin red according to established protocols (53). Specimens were first dehydrated and fixed in 95% ethanol. They were then incubated in Alcian blue staining solution for 1–2 days, followed by rinsing in 95% ethanol for 24 hours. Subsequently, specimens were cleared in 1% potassium hydroxide. The cleared bones were stained with alizarin red solution for 12–24 hours. After staining, specimens were further cleared through a graded series of 2% potassium hydroxide and glycerol mixtures (80:20, 60:40, 40:60, and 20:80, v/v). Finally, stained specimens were stored in glycerol.

### H&E staining.

Mice were transcardially perfused with ice-cold PBS followed by 4% paraformaldehyde in PBS. The whole brain was carefully dissected and postfixed in the same fixative for 24 hours at 4°C. Subsequently, the tissue was dehydrated through a graded ethanol series (75%, 85%, 90%, 95%, and 100%), cleared in xylene, and embedded in paraffin wax. Coronal sections of 2 μm thickness were cut using a rotary microtome (Leica). Dried paraffin sections were dewaxed in 2 changes of xylene, 20 minutes each, followed by immersion in 3 changes of 100% ethanol, 5 minutes each. After rinsing with water, the samples were stained with hematoxylin for 5 minutes, washed 3 times with water, and then differentiated with 1% hydrochloric acid ethanol for 5 seconds. This was followed by eosin staining for 5 seconds and gradient dehydration with 85%, 95%, and 100% ethanol. Finally, the samples were immersed in xylene twice, each for 3 minutes. The morphology and structure of the sections were observed using a whole-slide scanner VS200 (Olympus).

### Western blotting.

Tissue lysates were prepared in RIPA buffer (Beyotime Biotechnology) containing protease inhibitor cocktail (MilliporeSigma) and 1 mM PMSF. Protein concentrations were determined using the BCA Protein Assay Kit (P0012, Beyotime Biotechnology). A total of 20 μg sample mixture was then subjected to separation by SDS-PAGE at a constant voltage of 100 V, followed by transfer onto a PVDF membrane (MilliporeSigma). Next, the PVDF membranes were blocked with Protein-Free Rapid Sealing Solution (AR0041, Boster Biological Technology) at room temperature for 15 minutes and incubated with the primary antibody overnight at 4°C. On the following day, the PVDF membranes were incubated with secondary antibodies at room temperature for 2 hours. The protein signals were developed using the Enhanced Chemiluminescence kit (P10300, NCM Biotech) and captured with Image Lab software (Bio-Rad). Band intensity was normalized to β-actin using Image Lab software (Bio-Rad).

### Immunoprecipitation.

Tissues were homogenized on ice using immunoprecipitation lysis buffer containing a protease inhibitor cocktail (MilliporeSigma) and 1 mM PMSF. To analyze ubiquitination modifications, 10 mM *N*-ethylmaleimide was added to the lysis buffer. Protein concentrations were quantified using the BCA Protein Assay Kit (P0012, Beyotime Biotechnology) according to the manufacturer’s instructions. Preblocked protein G agarose beads were incubated with the target-specific antibody for 6 hours at 4°C. The antibody-bead complexes were then mixed with the lysates and incubated overnight at 4°C. Beads were pelleted by centrifugation and washed 5 times with cold lysis buffer. Bound proteins were eluted by boiling in loading buffer at 95°C for 15 minutes. Eluted proteins were resolved by SDS-PAGE and analyzed via Western blotting using relevant antibodies.

### Mass spectrometry and protein analysis.

Embryonic brain tissues were rapidly dissected, snap-frozen in liquid nitrogen, and stored at –80°C until shipment to Sangon Biotech Co., Ltd. for proteomics profiling. Briefly, samples were analyzed using a Q Exactive HF mass spectrometer (Thermo Fisher Scientific) integrated with an UltiMate 3000 RSLCnano liquid chromatography system (Thermo Fisher Scientific). Data analysis was performed using MaxQuant (version 1.6.6) software with the Andromeda search algorithm. The search was conducted against the mouse Proteome Reference Database for Mouse in UniProt.

### Statistics.

Animals were randomized into experimental groups. Investigators were blinded during both experimental procedures and outcome assessments to eliminate confirmation bias. Data were analyzed using GraphPad Prism (GraphPad Software) and are presented as the mean ± SEM in figures. The unpaired, 2-tailed Student’s *t* test was used to compare the means between 2 independent groups for continuous variables. One-way ANOVA was used for comparisons involving 3 or more independent groups. Two-way ANOVA was used for analyses involving 2 independent variables. Statistical significance was defined at a *P* value of less than 0.05.

### Study approval.

All experimental animal protocols were approved by the Soochow University IACUC.

### Data availability.

This manuscript does not report any original code. The mass spectrometry proteomics data have been deposited in the ProteomeXchange Consortium database (PXD072913). Figure 8A presents the re-analysis of RNA-seq data from the Gene Expression Omnibus (GEO) database (GSE256068). Values for all data points in graphs are available in the Supporting Data Values file.

## Author contributions

Xingshun Xu, HY, WG, and Xingyun Xu designed the research. Xingyun Xu, YZ, SX, XL, and YL performed the research experiments. Xingyun Xu, YZ, SX and HZ collected and analyzed data. YX helped with gene set enrichment analysis. ZM provided the reagents. Xingshun Xu and Xingyun Xu wrote the manuscript with comments from all authors. All authors approved the final version of the manuscript.

## Conflict of interest

The authors have declared that no conflict of interest exists.

## Funding support

- National Natural Science Foundation of China (82071511, to Xingshun Xu and 32400837, to Xingyun Xu).
- Jiangsu Provincial Science and Technology Program (SBE2023750299, to Xingshun Xu).
- Jiangsu Provincial Natural Science Foundation (BK20240362, to Xingyun Xu).
- China Postdoctoral Science Foundation (2024M762309, to Xingyun Xu).
- Postdoctoral Special Research Fund of The First Affiliated Hospital of Soochow University (202300470011, to Xingyun Xu).
- Jiangsu Funding Program for Excellent Postdoctoral Talent (2024ZB248, to Xingyun Xu).

## Supplementary Material

Supplemental data

Unedited blot and gel images

Supporting data values

## Version 1 05/15/2026

Electronic publication
