# Supplementary material for: Wdr26 insufficiency causes Skraban-Deardorff syndrome–like neurodevelopmental deficits in mice
Source: J Clin Invest. 2026 May 15;136(10):e195537. doi: 10.1172/JCI195537 (PMC13178648; doi:10.1172/JCI195537)
Supplement: Supplemental data [file jci-136-195537-s185.pdf]

Supplemental material

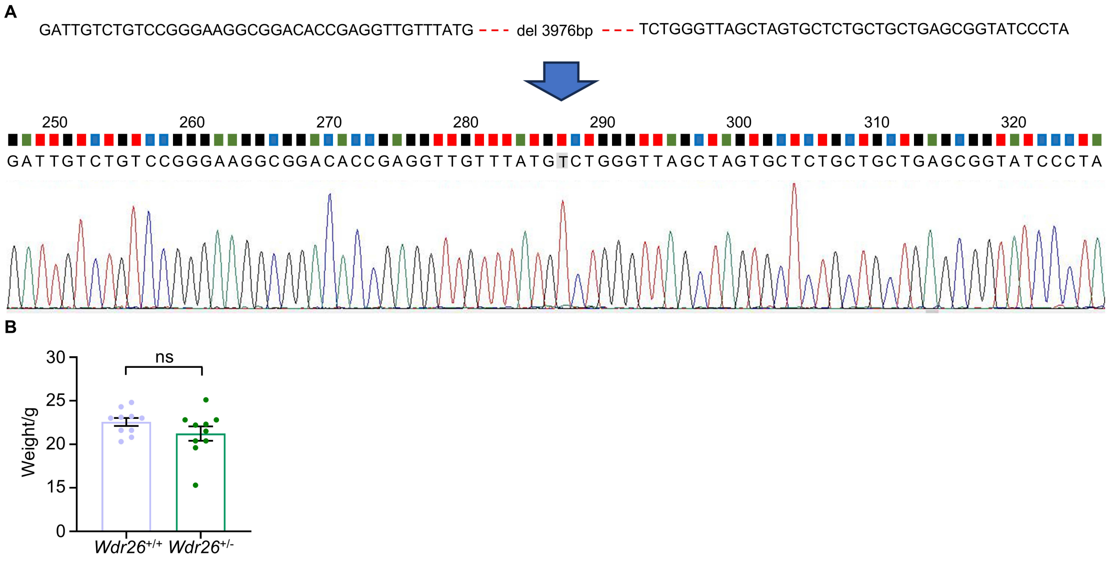

Supplementary Figure 1. Supplementary information on *Wdr26*$^{+/-}$ mice. (**A**) Schematic diagram of the *Wdr26* gene knockout strategy in transgenic mice. (**B**) Body weight of 2-month-old *Wdr26*$^{+/+}$ and *Wdr26*$^{+/-}$ mice. Statistical analysis was performed using unpaired two-tailed Student's t-test (ns, not significant).

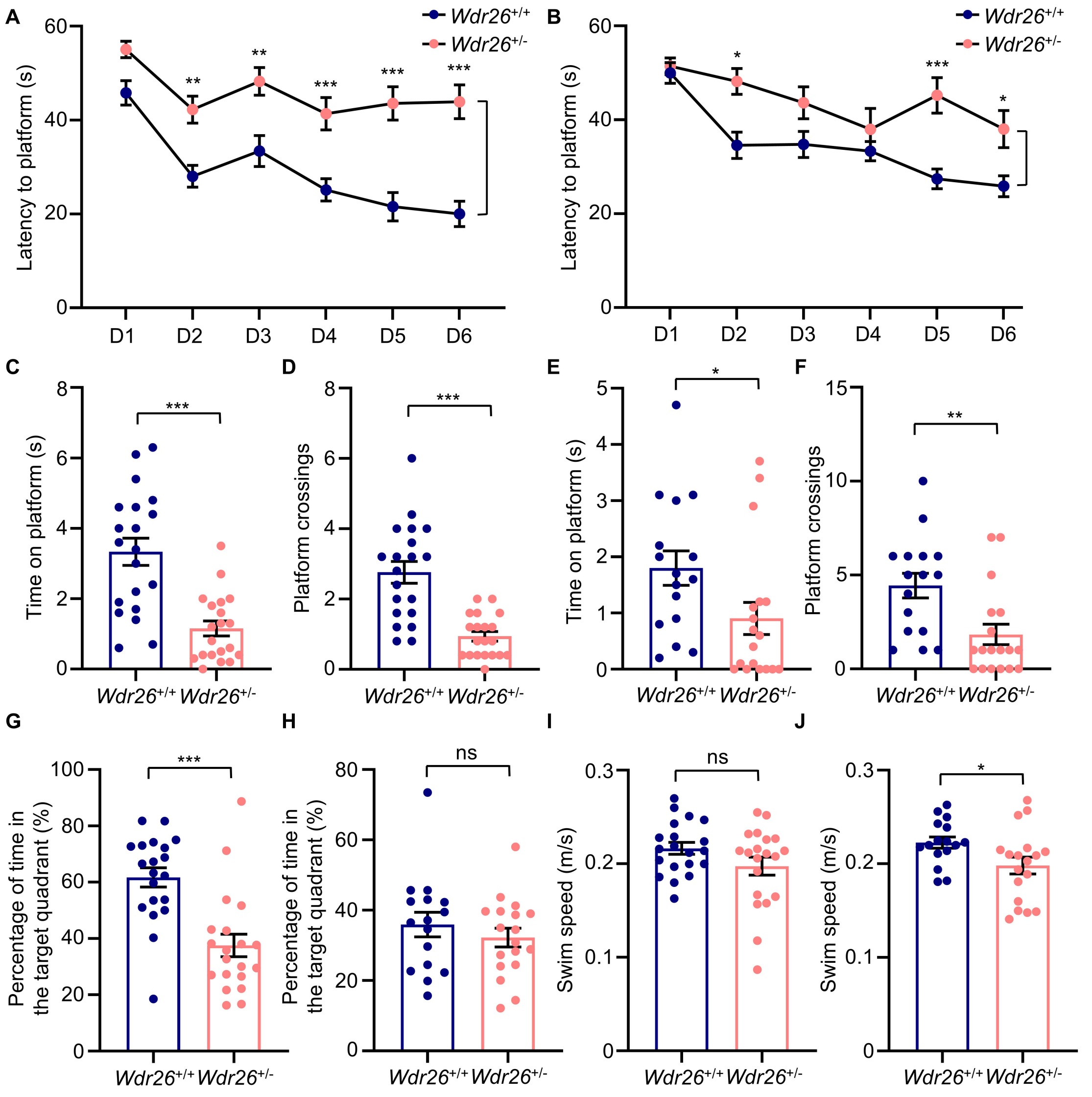

Supplementary Figure 2. Two-month-old *Wdr26*$^{+/+}$ and *Wdr26*$^{+/-}$ mice are tested in the MWM. (**A** and **B**) The escape latency in the training of MWM. Male (n = 20) (**A**), female (n = 16-18) (**B**). **(C)** The time spent on the platform of male *Wdr26*$^{+/+}$ and *Wdr26*$^{+/-}$ mice in the test of MWM (n = 20). **(D)** The platform crossings of male *Wdr26*$^{+/+}$ and *Wdr26*$^{+/-}$ mice in the test of MWM (n = 20). **(E)** The time spent on the platform of female *Wdr26*$^{+/+}$ and *Wdr26*$^{+/-}$ mice in the test of MWM (n = 16-18). **(F)** The platform crossings of female *Wdr26*$^{+/+}$ and *Wdr26*$^{+/-}$ mice in the test of MWM, (n = 16-18). (**G** and **H**) The percentage of time in the target quadrant in the test of MWM. Male (n = 20) (**G**), female (n = 16-18) (**H**). (**I** and **J**) The swim speed in

the test of MWM. Male (n = 20) (**I**) female, (n = 16-18) (**J**). Statistical analysis was performed using two-way analysis of ANOVA with Bonferroni's post hoc test for multiple comparisons (**A** and **B**) and unpaired two-tailed Student's t-test (**C**-**J**) (*p < 0.05, **p < 0.01, and ***p < 0.001 and ns, not significant).

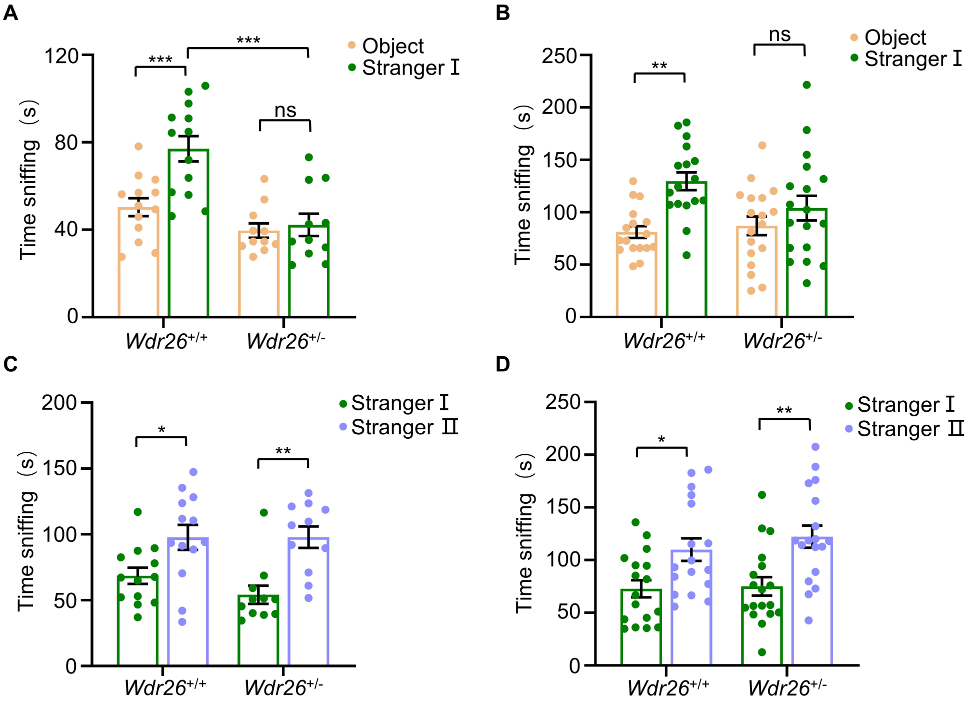

Supplementary Figure 3. Two-month-old *Wdr26*$^{+/+}$ and *Wdr26*$^{+/-}$ mice are tested in the three-chamber socialization test. (**A** and **B**) Quantification of investigative behaviors of *Wdr26*$^{+/+}$ and *Wdr26*$^{+/-}$ mice during phases I of the three-chamber socialization test was performed. Male (n = 11-13) (**A**), female (n = 17-18) (**B**). (**C** and **D**) Quantification of investigative behaviors of *Wdr26*$^{+/+}$ and *Wdr26*$^{+/-}$ mice during phases II of the three-chamber socialization test was performed. Male (n = 11-13) (**C**), female (n = 17-18) (**D**). Statistical analysis was performed using two-way ANOVA with Tukey's post hoc test for multiple comparisons. (*p < 0.05, **p < 0.01, and ***p < 0.001, ns, not significant).

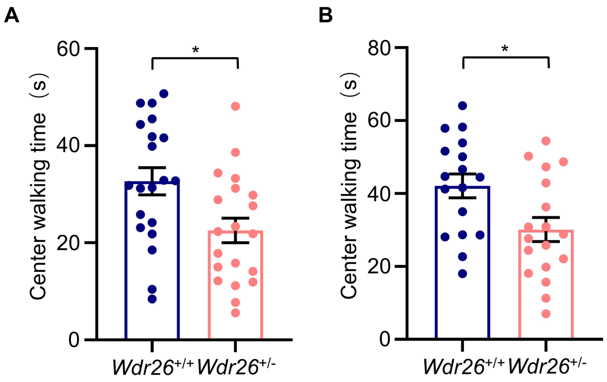

Supplementary Figure 4. Two-month-old *Wdr26*$^{+/+}$ and *Wdr26*$^{+/-}$ mice are tested in the open field test. (A and B) center walking time. Male, (n = 20) (**A**), female, (n = 17-18) (**B**). Statistical analysis was performed using unpaired two-tailed Student's t-test (*$p < 0.05$).

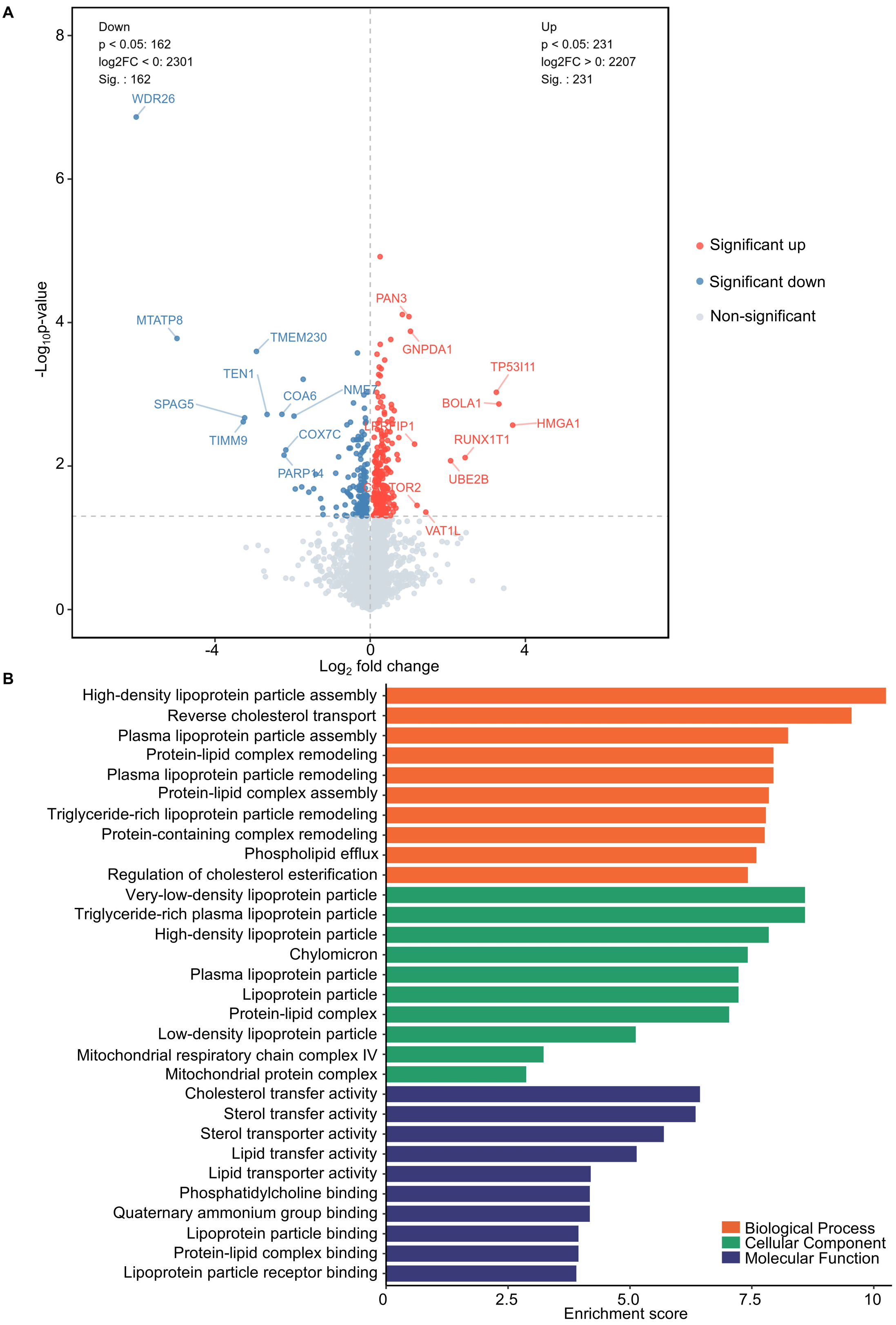

Supplementary Figure 5. Loss of *Wdr26* causes abnormalities in pathways related to lipid metabolism. (**A**) DEPs were shown in the volcano plots (n = 3). (**B**) The GO analysis of the

down-regulated proteins was shown.

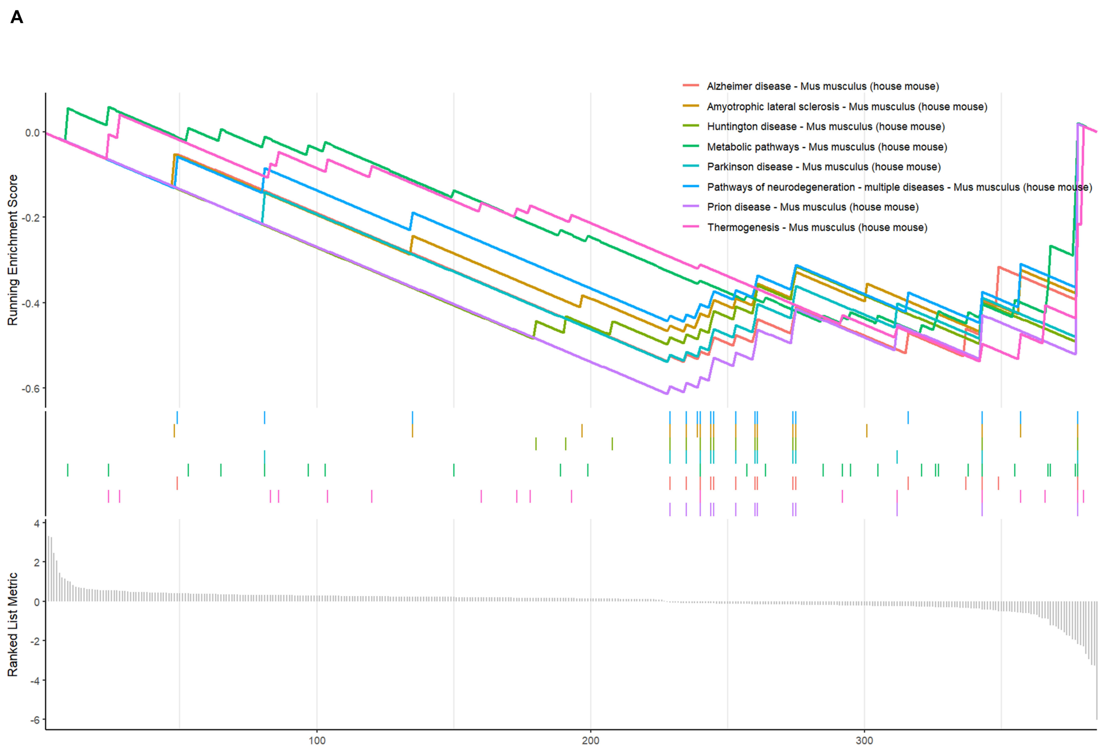

Supplementary Figure 6. Differentially expressed proteins induced by loss of *Wdr26* are linked to neurological disorders. (**A**) The GSEA analysis of the DEPs was shown.

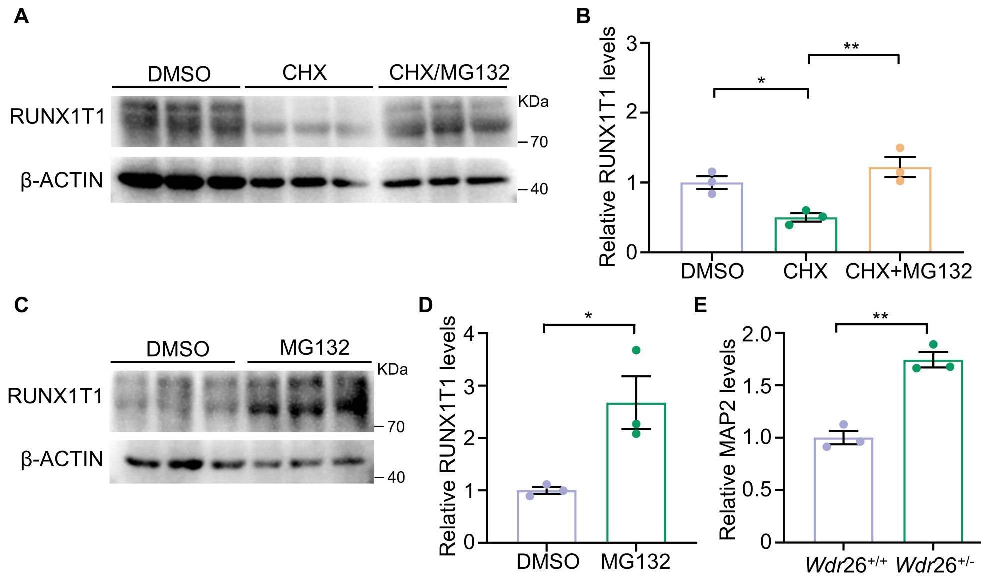

Supplementary Figure 7. The stability of RUNX1T1 is affected by the proteasome. (A)

N2a cells were treated with CHX and MG132 for 12 h and RUNX1T1 protein levels were tested by Western blotting. (**B**) Quantitative analysis of RUNX1T1 (A) protein levels was performed (n = 3). (**C**) N2a cells were treated with MG132 for 12 h and RUNX1T1 protein levels were tested by Western blotting. (**D**) Quantitative analysis of RUNX1T1 (**C**) protein levels was performed (n = 3). (**E**) Quantitative analysis of MAP2 (Figure 7**N**) protein levels was performed (n = 3). Statistical analysis was performed using one-way ANOVA with Tukey's post hoc test for multiple comparisons (**B**) and unpaired two-tailed Student's t-test (**D** and **E**) (*$p < 0.05$ and **$p < 0.01$).

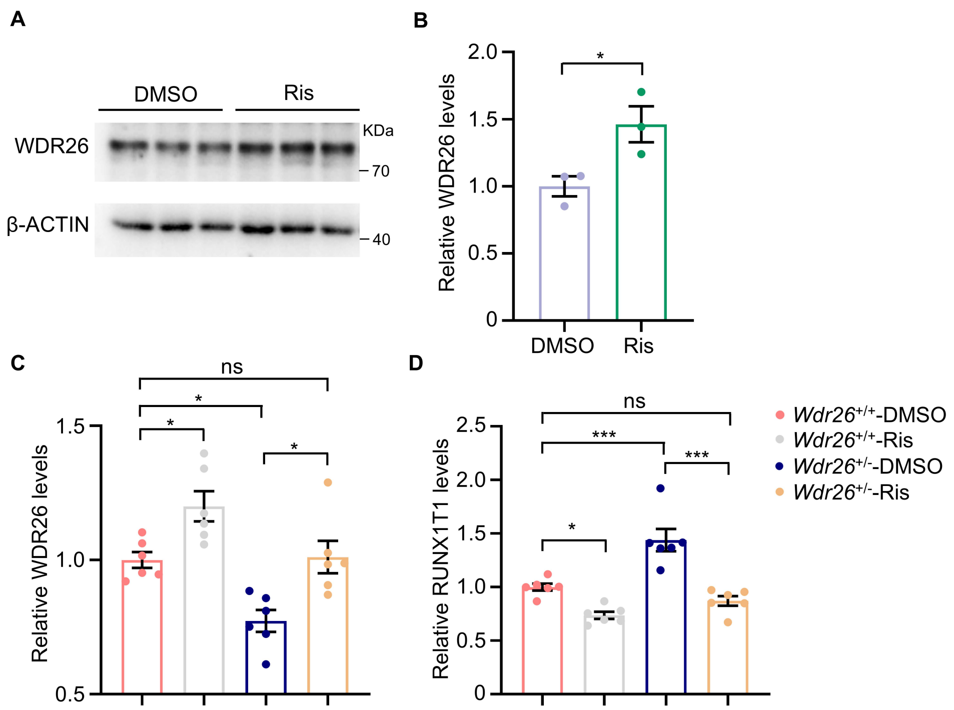

Supplementary Figure 8. The protein levels of WDR26 are increased by the risperidone. (**A**) HT22 cells were treated with 10 μM risperidone for 36 h and the protein levels of WDR26 were tested by Western blotting. (**B**) Quantitative analysis of WDR26 (**A**) protein levels was performed (n = 3). (**C**) Quantitative analysis of WDR26 (Figure 9**A**) protein levels was

performed (n = 6). Ris (risperidone). (**D**) Quantitative analysis of RUNX1T1 (Figure 9**B**) protein levels was performed (n = 6). Ris (risperidone). Statistical analysis was performed using unpaired two-tailed Student's t-test (**B**) and one-way ANOVA with Tukey's post hoc test for multiple comparisons (**C** and **D**) (*p < 0.05, ***p < 0.001 and ns, not significant).
