# Supplementary material for: Wdr26 insufficiency causes Skraban-Deardorff syndrome–like neurodevelopmental deficits in mice
Source: J Clin Invest. 2026 May 15;136(10):e195537. doi: 10.1172/JCI195537 (PMC13178648; doi:10.1172/JCI195537)

Full unedited gel for Figure 1B

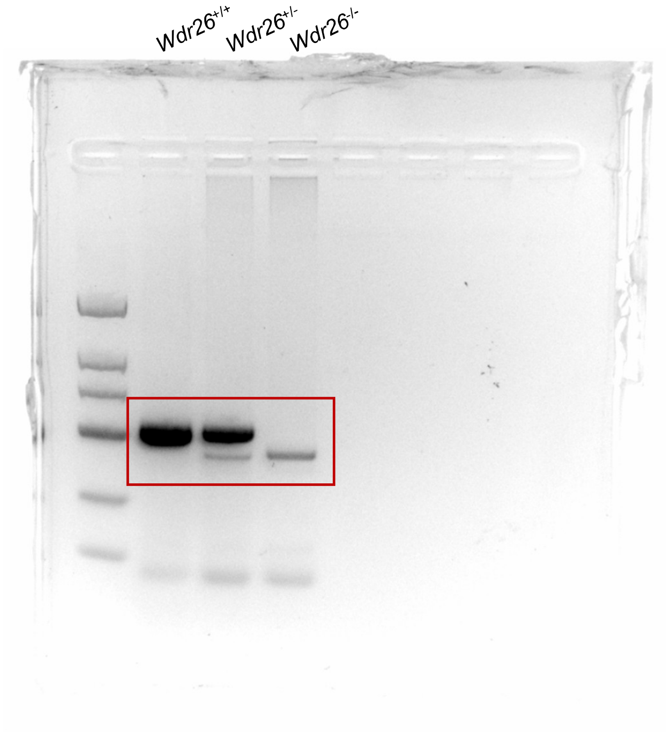

Full unedited blot for Figure 1E

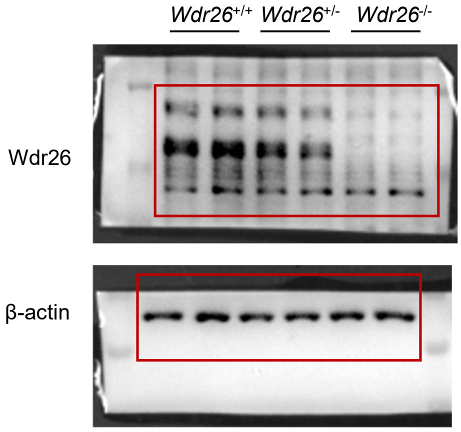

Full unedited blot for Figure 7

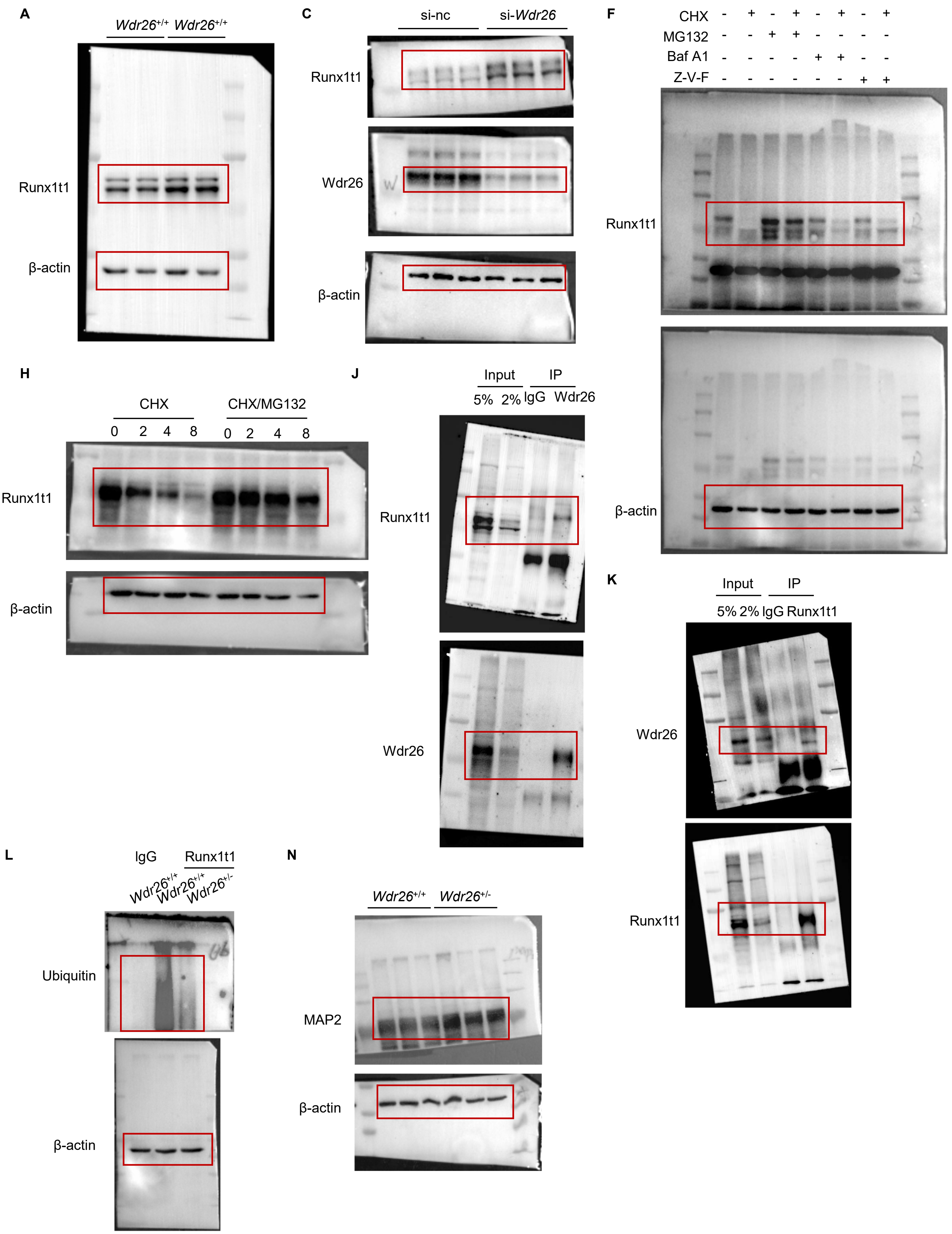

Full unedited blot for Figure 8

C

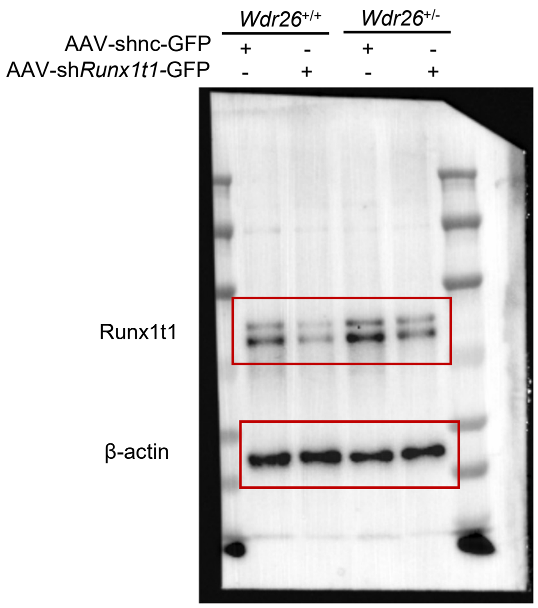

K

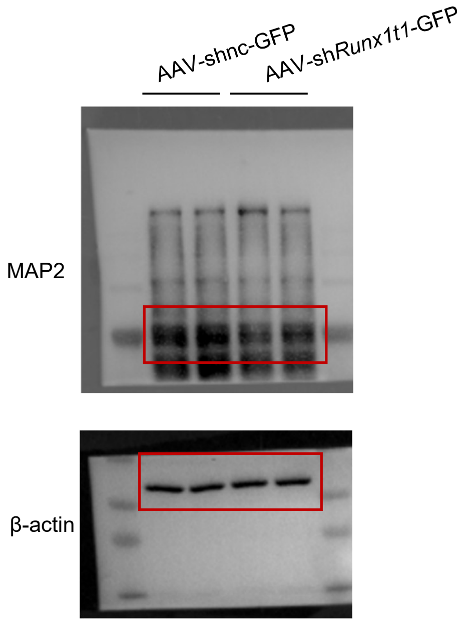

Full unedited blot for Figure 9

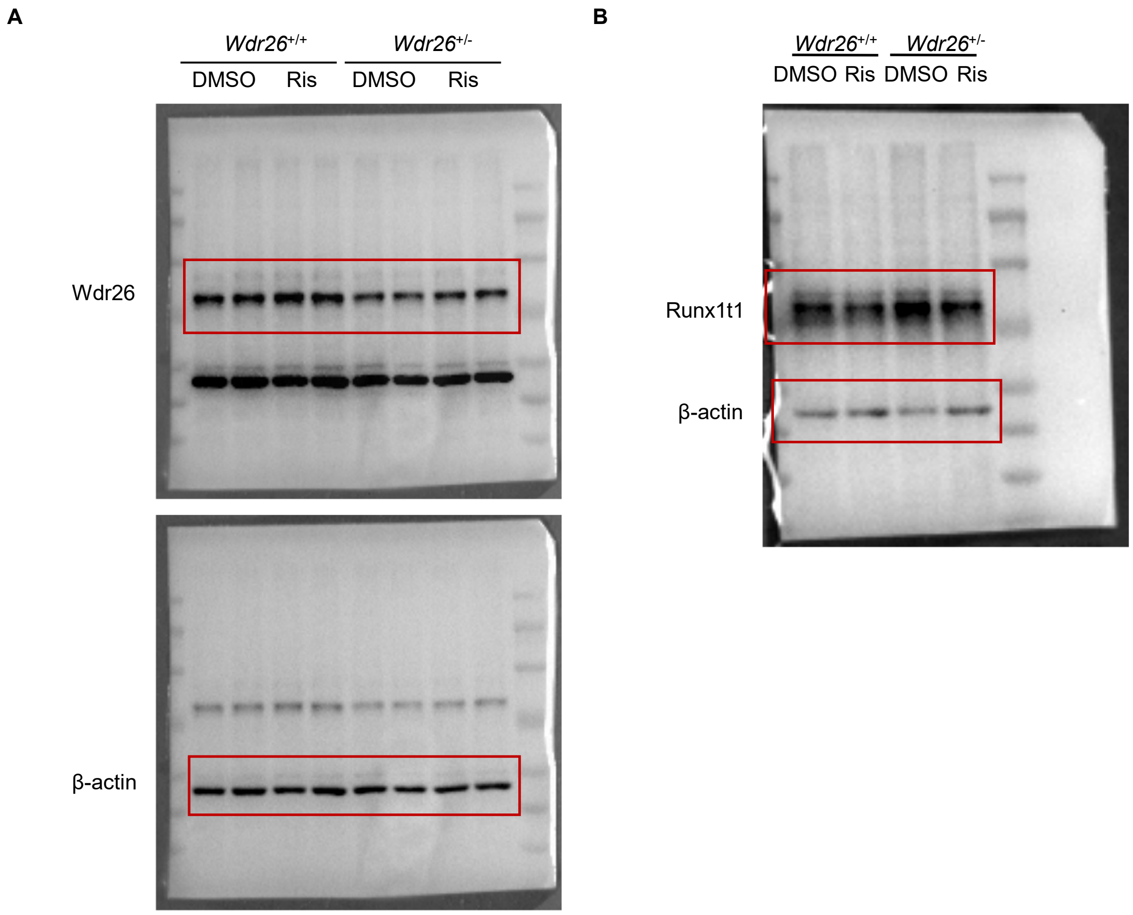

Full unedited blot for Supplemental Figure 7

A

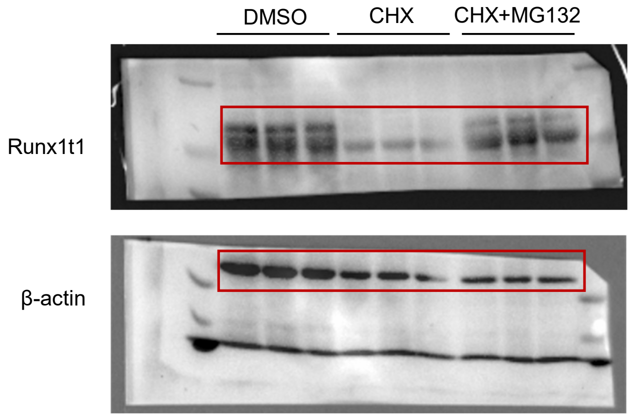

B

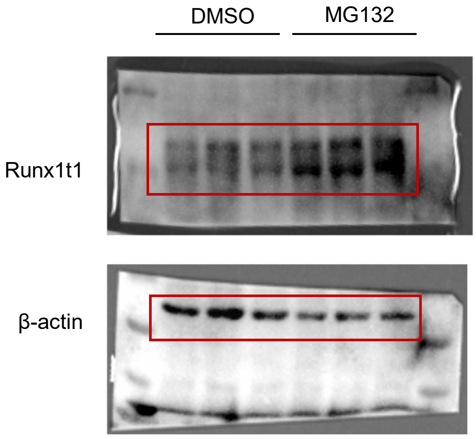

Full unedited blot for Supplemental Figure 8A

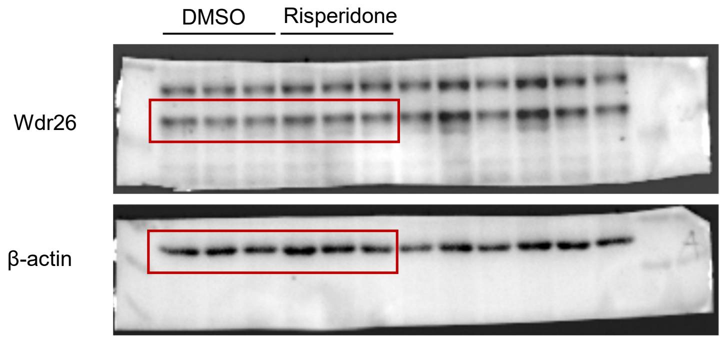

Supplement: Unedited blot and gel images [file jci-136-195537-s186.pdf]
